# Generating detailed intercellular communication patterns in psoriasis at the single-cell level using social networking, pattern recognition, and manifold learning methods to optimize treatment strategies

**DOI:** 10.18632/aging.205478

**Published:** 2024-01-29

**Authors:** Ying Xiong, Sidi Li, Yunmeng Bai, Ting Chen, Wenwen Sun, Lijie Chen, Jia Yu, Liwei Sun, Chijun Li, Jiajian Wang, Bo Wu

**Affiliations:** 1 Department of Dermatology, Shenzhen Maternity and Child Healthcare Hospital, Shenzhen 518028, China; 2 Institute of Pathology and Southwest Cancer Center, Southwest Hospital, Army Medical University (Third Military Medical University), Chongqing 400038, China; 3 Department of Nephrology, Shenzhen key Laboratory of Kidney Diseases, Shenzhen People’s Hospital, The First Affiliated Hospital, School of Medicine, Southern University of Science and Technology, Shenzhen 518055, Guangdong, China; 4 Department of Pediatrics, The Seventh Affiliated Hospital, Sun Yat-Sen University, Shenzhen 518107, Guangdong, China; 5 Scientific Research Center, The Seventh Affiliated Hospital, Sun Yat-Sen University, Shenzhen 518107, Guangdong, China; 6 Clinical Laboratory Department of The Second Affiliated Hospital, School of Medicine, The Chinese University of Hong Kong, Shenzhen and Longgang District People’s Hospital of Shenzhen, Shenzhen 518172, China; 7 Center for Energy Metabolism and Reproduction, Shenzhen Institute of Advanced Technology, Chinese Academy of Sciences, Shenzhen 518055, China; 8 Shenzhen Institute of Advanced Technology, Chinese Academy of Sciences, Shenzhen 518055, China; 9 Shenzhen Key Laboratory of Metabolic Health, Shenzhen 518055, China

**Keywords:** communication patterns, single cell transcriptome, cell type-specific regulons (CTSRs), proteomic sequencing, social networking

## Abstract

Psoriasis, a complex and recurrent chronic inflammatory skin disease involving various inflammatory cell types, requires effective cell communication to maintain the homeostatic balance of inflammation. However, patterns of communication at the single-cell level have not been systematically investigated. In this study, we employed social network analysis tools, pattern recognition, and manifold learning to compare molecular communication features between psoriasis cells and normal skin cells. Utilizing a process that facilitates the discovery of cell type-specific regulons, we analyzed internal regulatory networks among different cells in psoriasis. Advanced techniques for the quantitative detection of non-targeted proteins in pathological tissue sections were employed to demonstrate protein expression. Our findings revealed a synergistic interplay among the communication signals of immune cells in psoriasis. B-cells were activated, while Langerhans cells shifted into the primary signaling output mode to fulfill antigen presentation, mediating T-cell immunity. In contrast to normal skin cells, psoriasis cells shut down numerous signaling pathways, influencing the balance of skin cell renewal and differentiation. Additionally, we identified a significant number of active cell type-specific regulons of resident immune cells around the hair follicle. This study unveiled the molecular communication features of the hair follicle cell-psoriasis axis, showcasing its potential for therapeutic targeting at the single-cell level. By elucidating the pattern of immune cell communication in psoriasis and identifying new molecular features of the hair follicle cell-psoriasis axis, our findings present innovative strategies for drug targeting to enhance psoriasis treatment.

## INTRODUCTION

Psoriasis is a prevalent chronic inflammatory skin disease, affecting approximately 0.09% to 5% of the global population, yet its causes remain unclear [1–5]. Effective therapy is currently lacking, and relapses are frequent due to both external and internal factors, including psychological stress, bacterial infections, skin damage, and certain drug administrations (such as interferons, beta-blockers, and lithium) [2, 6–8]. The development of psoriasis is intricately linked to the activation, accumulation, and amplification of adaptive immune T cells, innate immune cells (neutrophils, macrophages, and dendritic cells), and resident skin cells (keratinocytes and endothelial cells).

In the pathogenesis of psoriasis, dying keratinocytes and those under stress release nucleic acids and antimicrobial peptides (AMPs, including LL-37), forming immunostimulatory complexes along with negatively charged RNA and free DNA [9–14]. These complexes activate plasmacytoid DCs (pDCs) and myeloid dendritic cells (DCs) via toll-like receptor 7 (TLR7) [12]. Activated plasma cells subsequently secrete type 1 interferons (IFN-α (Interferon Alpha 1), IFN-β), which, in turn, activate myeloid dendritic cells (mDCs) to produce cytokines such as IL-12 (Interleukin 12) and IL-23 (Interleukin 23) [12, 15, 16]. IL-23 also activates mDCs via toll-like receptor 8 (TLR8) [12]. These activated dendritic cells further stimulate T cell differentiation, and the resulting inflammatory cascades among resident immune cells contribute to the pathological changes observed in psoriasis [15, 16]. While extensive research has enhanced our understanding of the causes, progression, and treatment of psoriasis, there has been limited attention given to elucidating the primary modes of signaling among the various subclasses of psoriasis cells involved. Further studies are warranted to improve our comprehension of the underlying molecular biology and to develop new strategies for enhancing the treatment of psoriasis.

The scRNA-seq technique was employed to investigate molecular changes in major cell populations of psoriasis at the single-cell level and their roles in the inflammatory process [17–19]. However, studies on skin inflammation predominantly focus on identifying cell subpopulations, often neglecting their roles and interrelationships. For instance, Cheng et al. explored the transfer and proliferation of epidermal subpopulations among anatomical sites by comparing gene expression. They identified a subpopulation of “channel” keratinocytes with upregulated gap junction protein family genes and cation-transporting ATPase family genes [17]. Reynolds et al. proposed that interactions between endothelial cells and macrophages in inflammatory skin diseases recruit immune cells and help regulate immunity [18, 20]. While some studies concentrate on specific cell subsets, such as the enhanced circulation of memory CD8+ T cells in the skin of patients with psoriatic arthritis (PsA), considered responsible for dysregulating cutaneous immunity and inflammation development [21], or the significance of Tc17 cells (a subset of CD8+ T cells) and CXCL13 (C-X-C Motif Chemokine Ligand 13) in psoriasis [19], few have investigated the coordination of external communication patterns and internal regulatory networks among different cell subsets of psoriasis. This is especially true at the single-cell transcriptome level, where the focus should be on demonstrating how the genetic differences of various cell subtypes affect both internal and external regulatory relationships.

Cell types in solid tissues are not easily captured by scRNA-seq, and the process of suspension preparation is not conducive to collecting and culturing specific cellular taxa, such as neutrophil transcripts with low expression levels [22–24]. When identifying the role of different cell populations and cell communication in enacting immune responses, challenges arise in performing massively parallel sequencing to capture transcripts [25, 26], and the low expression of key cofactors such as affinity receptors and signaling molecules further complicates establishing cell communication patterns [25–27]. Nevertheless, the coordination of messaging among different immune cell subpopulations and intracellular regulatory networks plays a crucial role in the regulation of inflammation and is essential for dissecting the molecular signature of psoriasis. Improved understanding of intercellular communication patterns may enhance the design of new therapeutics, particularly in cell therapy. To address the coordinating relationship between the intracellular and extracellular components of psoriasis cells, we applied social network analysis tools, pattern recognition methods, and manifold learning approaches to explore communication patterns between cells [28–31]. Utilizing pharmacogenomic data, we identified cell type-specific regulons (IRIS3) involved in the internal regulatory network among different cells in psoriasis. This discovery provides additional evidence for the interconnectedness of the internal regulatory network and external communication patterns, highlighting prospective targets for therapeutic interventions in the hair follicle-psoriasis axis [32, 33]. Furthermore, we employed the most advanced protein pathological section detection technology to identify proteins within a restricted set of diagnosed tissue sections. Subsequently, we established the correlation between the communication patterns of psoriasis cells and their clinical significance. Additionally, we confirmed the validity of the hair follicle axis hypothesis through the examination of pathological specimens [34–37].

## RESULTS

### Differences in cell communication between psoriatic and normal skin

**Figure 1:**
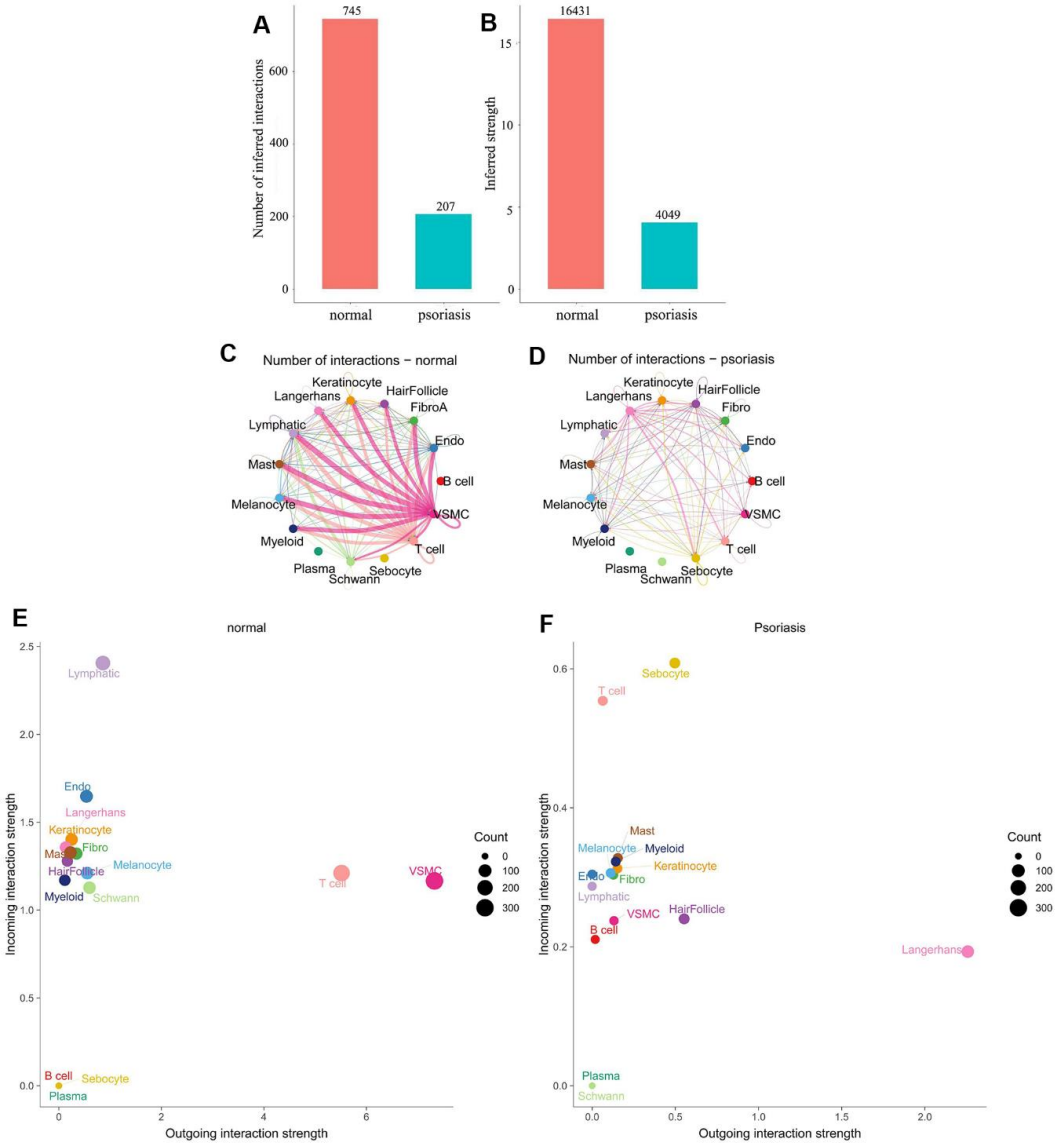
**Differences in cell subpopulation communication between psoriatic and normal skin.** (**A**, **B**) Differences in the number and intensity of communications. (**C**, **D**) Interaction relationships for each cell subtype. (**E**, **F**) Differences in signal input and signal output patterns.

To investigate and compare cell-cell communication in psoriasis and healthy skin, we gathered single-cell transcriptomic datasets from 5 patients with psoriasis and 3 normal skin volunteers. Cells were categorized into 15 types (Figure 1) based on literature protein markers: B-cells, T-cells, fibroblasts, hair follicles, keratinocytes, Langerhans cells, lymphatic endothelial cells, mast cells, melanocytes, myeloid cells, plasma cells, and Schwann cells. Cellchat was employed for the analysis of scRNA-seq datasets from psoriatic and normal skin.

Comparing cellular communication intensity between psoriasis and normal skin revealed a significant quantitative difference, with 207 for psoriasis and 745 for normal skin (Figure 1A, 1B). Analysis of the interacting cell types demonstrated a positive correlation between the number and intensity of cell communications, but the primary cell populations involved varied significantly between the two states. Normal skin was characterized by a dominance of VSMC (Vascular smooth muscle cells) and T-cells, while psoriatic skin exhibited dominance by Langerhans cells (Figure 1C, 1D).

Further examination of the input and output patterns of communication indicated that the intensity of output signals was primarily dominated by VSMC and T-cells in normal skin, whereas Langerhans cells were the predominant output signals in psoriasis. Sebocytes and T-cells displayed higher intensity in the input signal pattern of psoriatic skin, while lymphatic cells were dominant in normal skin (Figure 1E, 1F). Additionally, we observed relatively weak input and output signals of plasma cells in both physiological states, even though their signaling pathways were active. Weak signals were also generated in normal skin by B-cells and sebocytes, while in psoriasis, certain cell types, notably plasma and Schwann cells, either produced weak signals or no signals at all. Notably, changes in the communication pattern of immune cells in the pathological state were identified: B-cells were activated, and T-cells shifted from predominantly being output signals to predominantly being input signals (Figure 1E, 1F). These findings underscore significant differences in the number of interactions and cell populations between normal and psoriatic skin.

### Information flow in psoriatic and normal skin

**Figure 2:**
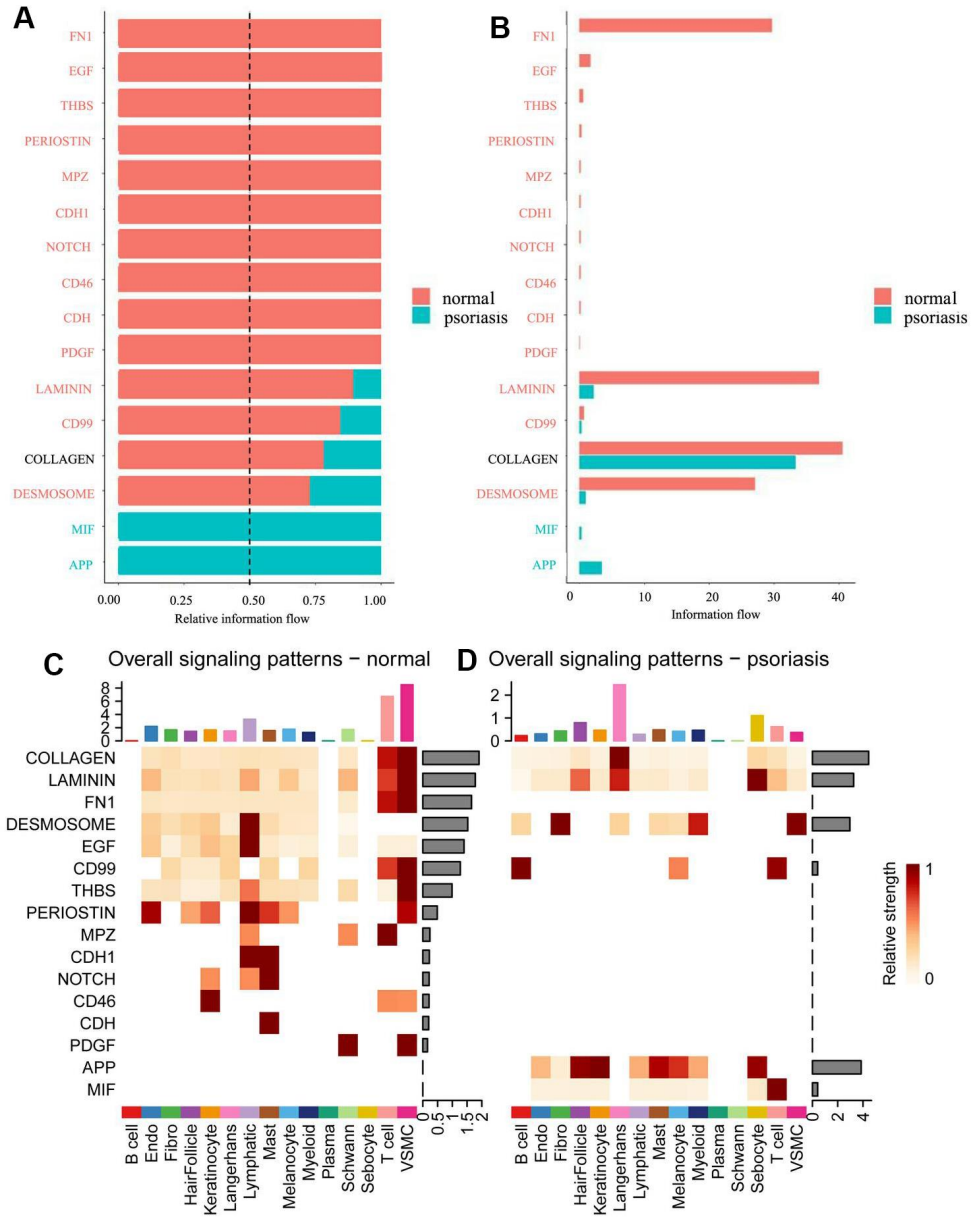
**Comparison of information flow between psoriatic and normal skin.** (**A**) Relative information flow. (**B**) Information flow. Red represents normal skin, blue represents psoriasis. (**C**) Global signal distribution of each subtype of cells in normal skin. (**D**) Global signal distribution of each subtype of cells in psoriatic skin.

Differences in cellular information flow between psoriatic and normal skin were calculated based on the sum of communication probabilities between cell populations (Figure 2A, 2B). Our results revealed significant disparities in information flow between psoriatic and normal skin: normal skin exhibited activity in ten signaling pathways (FN1 (Fibronectin 1), EGF (Epidermal Growth Factor), THBS (Thrombospondin 1 pathway), PERIOSTIN (Periostin), MPZ (Myelin Protein Zero), CDH1 (Cadherin 1-mediated signaling pathway), NOTCH (Notch signaling pathways), CD46 (Trophoblast-Lymphocyte Cross-Reactive Antigen), CDH, PDGF (Platelet-Derived Growth Factor)), while psoriasis skin presented two unique signaling pathways (MIF (Macrophage Migration Inhibitory Factor) and APP (Amyloid-Beta Precursor Protein)). Unexpectedly, we identified several communication pathways in normal skin that were relatively more active than psoriatic pathways, including LAMININ (LAMC1-mediated signaling pathways), CD99 (Cell Surface Antigen 12E7), COLLAGEN (Collagen), and DESMOSOME (Desmosome).

Different cell types in different skin states were found to activate distinct signaling or communication pathways (Figure 2C, 2D and Supplementary Figures 1, 2). In normal skin, the primary signaling pathways for T-cells and VSMC included COLLAGEN, LAMININ, FN1, CD99, THBS, PERIOSTIN, MPZ, and PDGF (Figure 2C). In psoriasis, Langerhans cells were predominantly and strongly involved via COLLAGEN and LAMININ pathways (Figure 2D), while a variety of cell types participated in the APP pathway, primarily featuring T-cells in the MIF pathway.

### Identification of critical proteins involved in signaling pathways by proteomic sequencing of pathological sections

**Figure 3:**
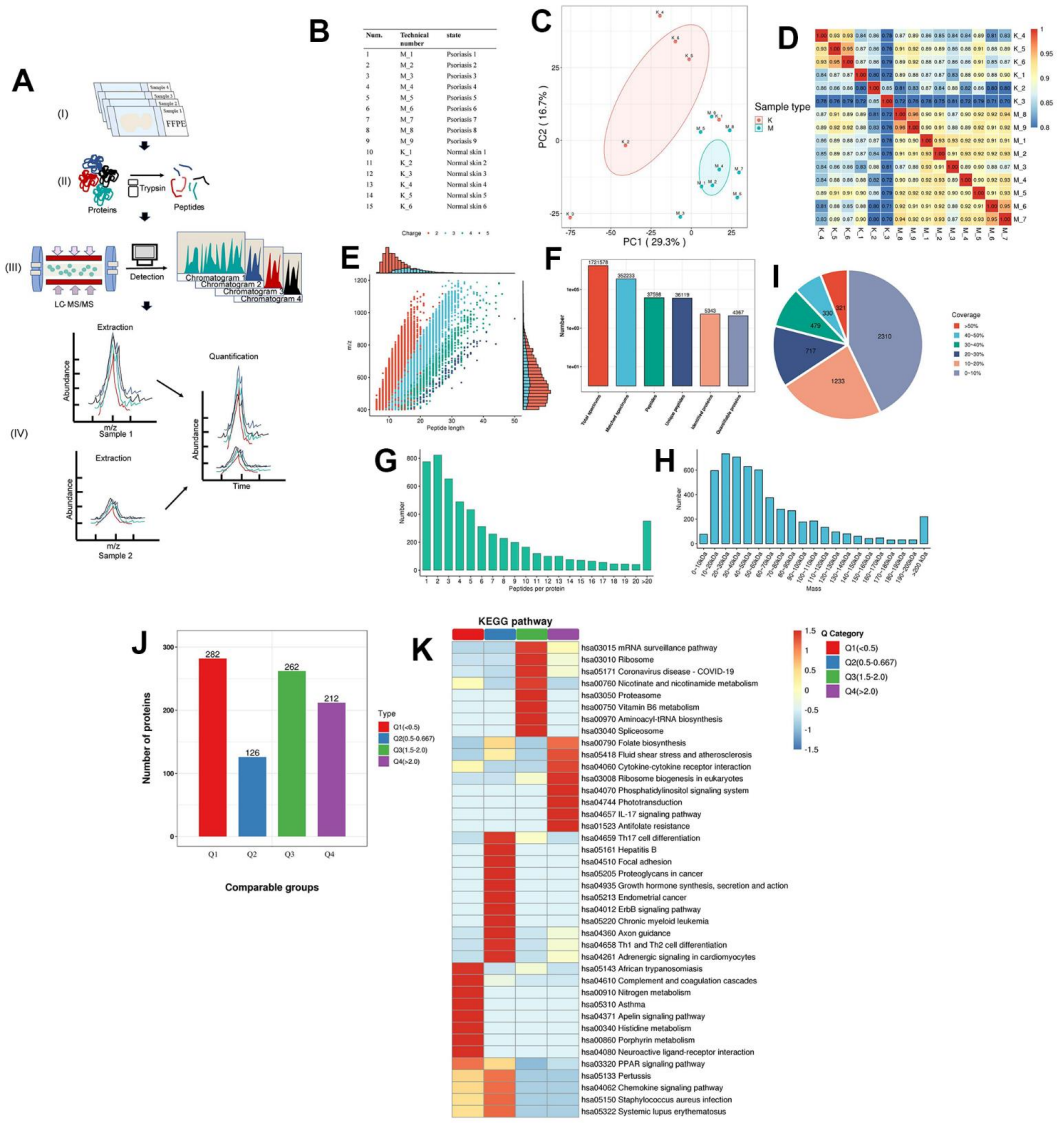
**Proteomics of pathological tissue sections in psoriatic skin and normal skin.** (**A**) Schematic diagram of the quantitative proteomics sequencing process for formalin-fixed and paraffin-embedded (FFPE) pathology sections. (I) Formalin-fixed and paraffin-embedded (FFPE) pathology section. (II) Enzymatic digestion and extraction of proteins. (III) Schematic diagram of LC-MS/MS. (IV) Label-free quantification. (**B**) Clinical information on patients: 9 patients with psoriasis and 6 specimens of normal skin. (**C**) Principal component cluster analysis for protein quantification of all clinical samples, with the degree of aggregation representing the magnitude of inter-sample variability. The results show that the normal and psoriatic samples clustered to form two major groups, indicating the heterogeneity that exists between the samples and a true reflection of the differences between the two groups. (**D**) Correlation analysis between all samples. The colours and numbers represent the strength of the correlation. (**E**) Peptide length distribution in pathological histological sections of psoriatic skin and normal skin. The vertical axis represents the ratio of charged particle mass to charge, i.e. the mass-to-charge ratio (m/z), and the horizontal axis represents the peptide length, with each point indicating a different peptide segment and the colour indicating a different charge gradient. The bar graph represents the charge. (**F**) Protein identification analysis. Total spectrums: the number of secondary spectra generated by mass spectrometry. Matched spectrums: the number of valid spectra, the number of spectra matching the theoretical secondary spectrum. Peptides: the number of identified peptides, the number of peptide sequences resolved by matching. Unique peptides: the number of identified unique peptides, the number of unique peptide sequences resolved by matching. Identified proteins: number of identified proteins, number of proteins resolved by specific peptides. Quantifiable proteins: number of proteins quantified by specific peptides. (**G**) Distribution of the number of peptides. (**H**) Protein molecular weight distribution. (**I**) Protein coverage distribution. Most proteins have less than 30% coverage. In a shotgun (bottom-up) technique based on mass spectrometry, the mass spectra scan for peptides with a greater abundance preference. Thus, protein coverage and abundance in the sample are positively correlated. (**J**) Quality classification of differential proteins and their KEGG analysis. The quality of differentially expressed proteins is divided into four levels (see Supplementary Table 7 for details). Light purple indicates Q1, light green indicates Q2, pink indicates Q3, light blue indicates Q4. (**K**) The KEGG pathway of the differential proteins after classification into different groups. Dark red indicates the level of pathway activity. The classical IL-17 pathway is observed in the higher quality Q4 group, which also contains cytokine receptor interactions, further supporting the molecular hypothesis of the follicular psoriasis axis.

To scrutinize the expression of a diverse array of critical proteins implicated in signaling pathways, we conducted quantitative proteomic analysis on formalin-fixed and paraffin-embedded (FFPE) skin samples obtained from 9 patients with psoriasis and 6 normal patients, employing state-of-the-art FFPE quantitative proteomics technology (Figure 3A). All specimens, both from psoriatic and normal skin, underwent characterization by two protein groupings through principal component analysis (PCA) (Supplementary Table 1 and Figure 3B, 3C). Pearson’s r values consistently exceeded 0.70 across all specimens, with a correlation of 0.92 observed between independently prepared tissues from the same psoriasis patient (Figure 3D). The majority of peptides in the specimens comprised 7-20 amino acids, aligning with the general pattern obtained through enzymatic digestion and mass spectrometry fragmentation. The distribution of peptide lengths identified by mass spectrometry adhered to quality control requirements (Figure 3E). Employing a recently described, highly sensitive label-free proteomic workflow capable of accurately quantifying most cellular proteomes, we identified and quantified over 5000 proteins (Figure 3F and Supplementary Table 5) [35, 37]. Permutation-based false discovery rate (FDR) analysis was executed to correct for multiple hypothesis testing in the Proteome Discoverer (v2.4.1.15) environment [38]. Additionally, we observed that the molecular weights of the identified proteins varied at different stages and were evenly distributed (Figure 3G). Most proteins corresponded to more than two peptides, with 10 or fewer defining the majority (Figure 3H). Our monitoring further revealed that the majority of proteins had less than 30% coverage (Figure 3I). In shotgun (also known as bottom-up) based mass spectrometry methods, spectra are preferentially scanned for peptides of higher abundance, suggesting a positive correlation between protein coverage and abundance in the samples.

We observed the detectability of most proteins involved in the aforementioned information pathways in psoriatic skin (Supplementary Table 6 and Supplementary Figures 3, 4), affirming the clinical significance of our results. Additionally, the molecular communication status of psoriatic skin was found to be similar between Caucasian and Chinese groups, providing an advantage for targeted drug development and patient treatment. Moreover, through a comprehensive comparison of globally differentially expressed proteins, categorized into four classes based on the fold of differential expression, we identified interactions among cytokines and receptors beyond the classical IL-17 pathway in psoriasis (Figure 3J, 3K and Supplementary Tables 7, 8, 11–14). These experimental findings underscore that the utilization of pathological sections yielded results consistent with our overarching analysis.

Examining the results of information flow and signaling patterns (Figure 2), it is evident that numerous signaling pathways in normal skin are deactivated in pathological skin. Notably, signaling pathways such as NOTCH and PERIOSTIN, crucial for the renewal of differentiation homeostasis in skin cells, exhibited reduced expression of NOTCH receptors associated with atopic dermatitis [39]. In our investigation of pathological tissue sections of psoriatic skin, we observed very low expression of NOTCH-related proteins, including JAG1 (Jagged Canonical Notch Ligand 1), JAG2 (Jagged Canonical Notch Ligand 2), and NOTCH2 (Neurogenic locus notch homolog protein 2) (Supplementary Table 5). JAG1 and JAG2 were not detected, while NOTCH2 was present in very low abundance. POSTN, responsible for encoding periosteal proteins and collagen expression in the skin, often interacts with ITGAV (Integrin Subunit Alpha V) and ITGB5 (Integrin Subunit Beta 5) between fibroblasts [40]. In normal skin, we observed a higher signal intensity in multiple cell types within the PERIOSTIN pathway compared to psoriasis. Subsequently, we confirmed the upregulation of PERIOSTIN pathway-related protein POSTN expression in pathological sections (Supplementary Table 5; Psoriasis/Normal skin Ratio in POSTN protein: 0.813 (or Normal/Psoriasis skin Ratio in POSTN protein: 1.230), Psoriasis/Normal skin Ratio in ITGAV protein: 0.938 (or Normal/Psoriasis skin Ratio in ITGAV protein: 1.066), Psoriasis/Normal skin Ratio in ITGB5 protein: 0.614 (or Normal/Psoriasis skin Ratio in ITGB5 protein: 1.629)). The involvement of multiple cell types in psoriasis, particularly in the more frequent APP pathway, was further confirmed at the protein level. We identified APP pathway-related ligand receptor proteins, including APP and CD74 (CD74 Antigen), with upregulated protein expression in pathological tissue sections of psoriatic skin (Supplementary Table 5; Psoriasis/Normal skin Ratio in CD74 protein: 1.202; Psoriasis/Normal skin Ratio in MIF protein: 1.239).

### Changes in communication patterns in multiple cell subtypes of psoriasis

**Figure 4:**
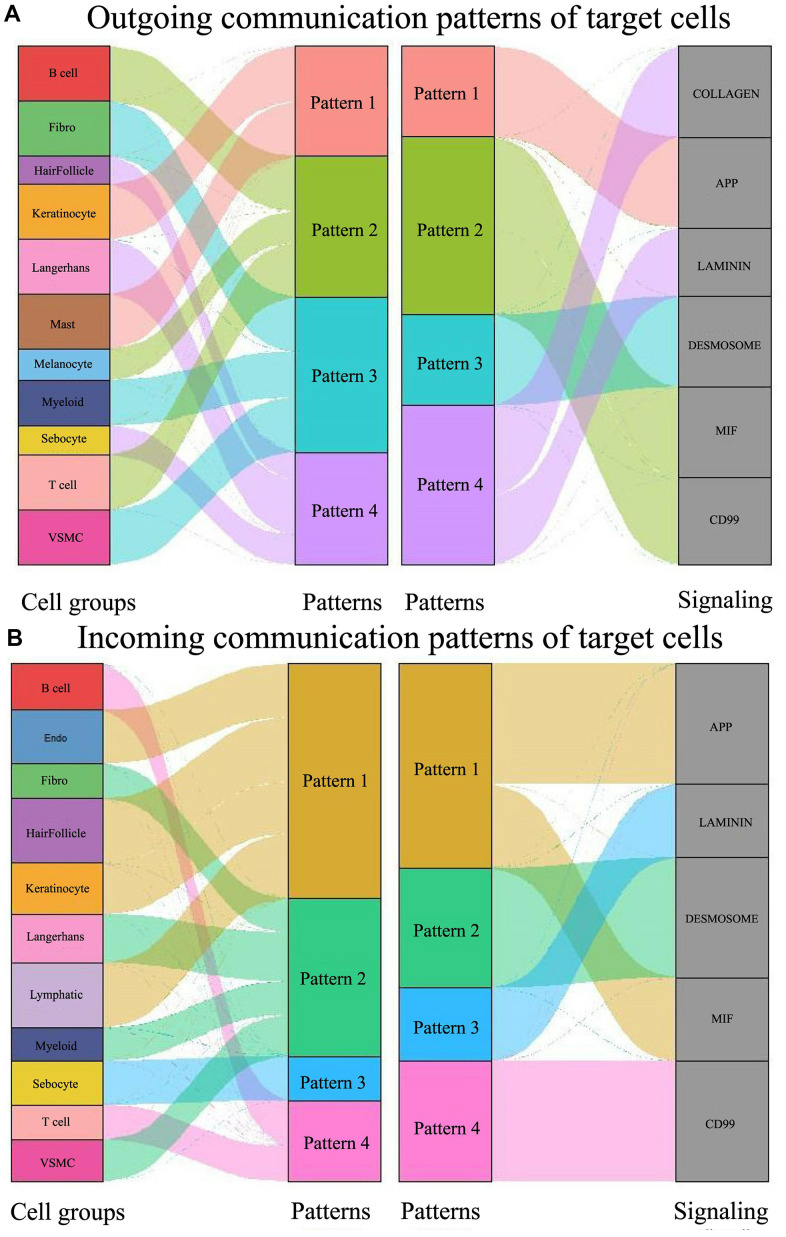
**Global signal pattern of each subtype of cells in psoriasis.** (**A**) Signal outgoing pattern. (**B**) Signal incoming pattern.

The pattern recognition approach developed in this study enables the linkage of cell populations and signaling pathways to discern different groups of cells as signal senders or receivers. We identified four outgoing signaling patterns and four incoming signaling patterns as follows (Figure 4):

1. Outgoing signaling pattern #1 is the APP pathway, predominantly led by keratinocytes and mast cells. When pattern #1 transitions to the incoming pattern, the APP pathway is reinforced by the MIF pathway. The cell types involved are presumed to shift to endothelial, hair follicle, keratinocyte, and lymphatic.
2. Outgoing pattern #2 involves B-cells, T-cells, and melanocytes, corresponding to the MIF and CD99 signaling pathways, typical in the immune process. In the incoming patterns, B-cells and T-cells fall under pattern #4, which exclusively involves CD99 signaling. Here, the participating cells of outgoing pattern #2, in turn, engage in incoming pattern #4.
3. Fibro, Myeloid, and VSMC constitute outgoing pattern #3, encompassing the DESMOSOME signaling pathway. Fibro, Myeloid, and VSMC incorporate Langerhans when transitioning to input signaling pattern #2. Here, the participating cells of outgoing pattern #3, in turn, participate in incoming pattern #2.
4. Hair follicles, Langerhans, and sebocytes contribute to outgoing pattern #4 involving COLLAGEN and LAMININ. When shifted to the input pattern, these three types of cells are engaged in three different incoming signaling pathways: the hair follicle participates in incoming pattern #1, Langerhans in incoming pattern #2, and sebocytes in incoming pattern #3. Here, the participating cells of outgoing pattern #4, known for their flexibility, are again involved in incoming pattern #1, pattern #2, and pattern #3.

These results illustrate that the signaling pathways of different cell types in psoriasis form diverse yet relatively stable communication patterns, identifiable as incoming and outgoing processes. One cell type may synergize with one or more other cell types in one or more pathways to fulfill its role in the pathogenesis of psoriasis. Additionally, we observed that certain cell types, such as plasma cells and Schwann cells, are not implicated in intercellular communication in psoriasis (Figures 1E, 1F, 2C, 2D and Supplementary Figures 1, 2).

### The role of hair follicle cells in intercellular communication in psoriasis

**Figure 5:**
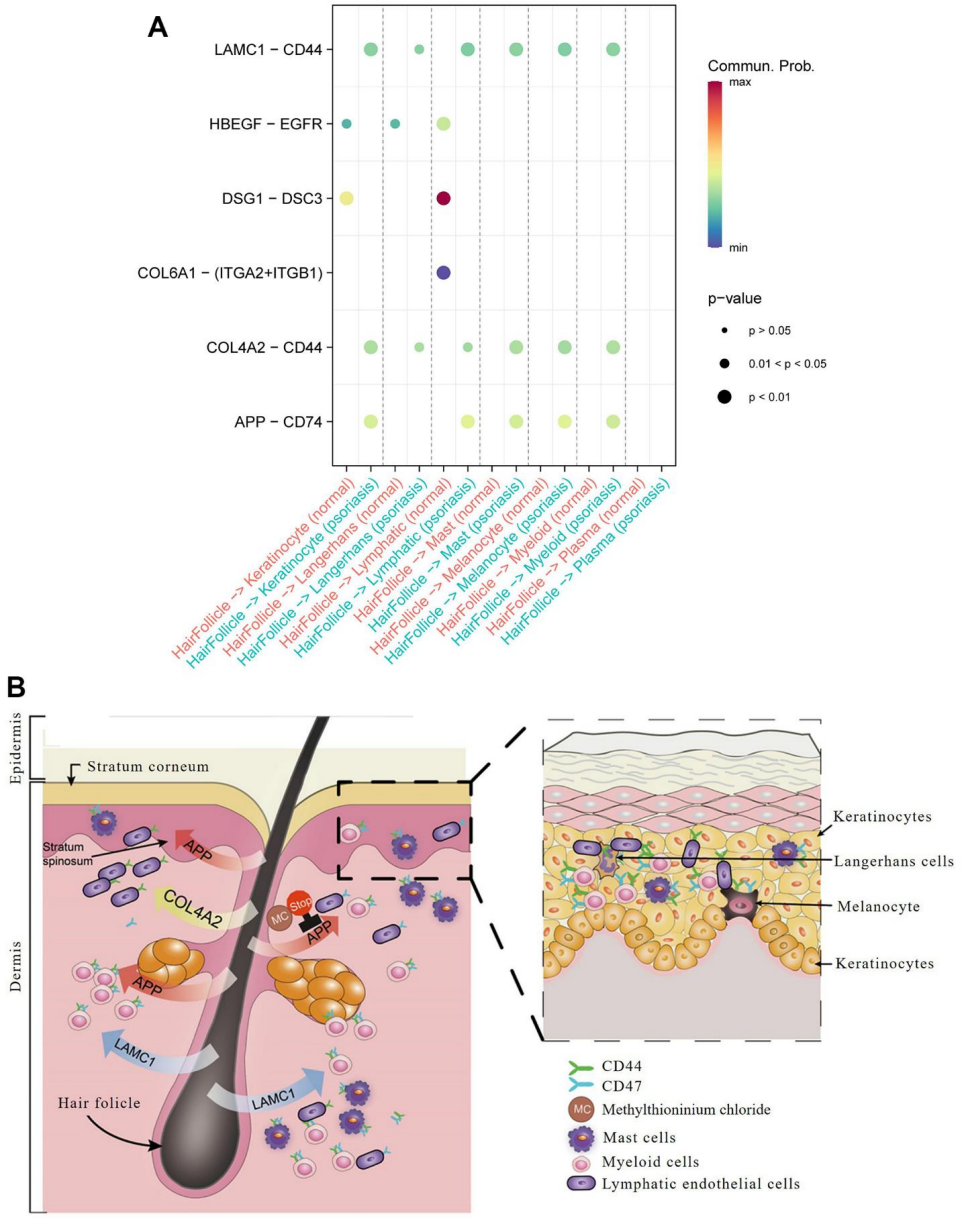
**Hair follicle-psoriasis axis at the single cell level.** (**A**) Significant ligand-receptor pairs between different subtypes of cells in psoriatic and normal skin. The size of the circles represents significance, and the colour represents the magnitude of the probability of communication between subtypes of cells. (**B**) Diagram of the “hair follicle-psoriasis axis” at the single cell level. Hair follicles release APP, LAMC1 or COL4A2, which communicates with resident immune cells such as melanocytes, mast cells and myeloid cells, thereby activating the immune “garrison” in the local area.

Hair follicle cells are widely recognized as the immune sentinels of human skin [6]. Our data corroborate their ability to communicate with a diverse range of cell types (Figure 5) and reveal distinct differences between normal and psoriatic skin in cellular communication pathways and the involvement of ligand-receptor pairs (Supplementary Figures 3, 4 and Supplementary Table 6).

1. Communication between hair follicle cells and keratinocytes was found to be more robust in psoriasis than in normal skin, with entirely different ligand-receptor pairs: DSG1 (Desmoglein 1) / DSC3 (Desmocollin 3) and HBEGF (Heparin Binding EGF Like Growth Factor) / EGFR (Epidermal Growth Factor Receptor) in normal skin, and APP/CD74, COL4A2 (Collagen Type IV Alpha 2 Chain) / CD44 (Cell Surface Glycoprotein CD44), and LAMC1 (Laminin Subunit Gamma 1) /CD44 in psoriasis.
2. Communication between hair follicle cells and Langerhan cells was associated with COL4A2/CD44 and LAMC1/CD44 in psoriasis, contrasting with a relatively weak HBEGF/EGFR ligand-receptor pair in normal skin.
3. Communication between hair follicles and lymphatic endothelial cells revealed prominent ligand-receptor pairs in normal skin, such as DSG1 and its receptor DSC3, COL6A1 (Collagen Type VI Alpha 1 Chain) and its receptors ITGA2 (Integrin Subunit Alpha 2) and ITGB1 (Integrin Subunit Beta 1), while in psoriasis, they were APP and its receptor CD74, LAMC1 and its receptor CD44, and COL4A2 and its receptor CD44.
4. Communication between hair follicles and mast cells, melanocytes, and myeloid cells only occurred in psoriasis, focusing on COL4A2/CD44 and APP/CD74 ligand-receptor pairs.

These results underscore the central role of hair follicles in psoriasis through their interaction with resident immune cells. Hair follicles exhibited heightened communication with immune cells in the superficial skin layer, including keratinocytes and endothelial cells, in terms of ligand-receptor relationships. Notably, we found that virtually all ligand receptors involved in the activity of hair follicles and other cell types could be detected in tissue sections of psoriasis patients stored for many years, such as CD74 (Psoriasis/Normal skin Ratio in CD74 protein: 1.202) and CD44 (Psoriasis/Normal skin Ratio in CD44 protein: 1.230) (Supplementary Figures 3, 4 and Supplementary Tables 5, 6). Subcellular localization analysis revealed their predominant concentration extracellularly, further validating their role in intercellular interactions (Figure 5 and Supplementary Table 5). This finding holds significance for the collection and preservation of clinical specimens, patient diagnosis, and treatment.

Based on the above analysis, we conclude that LAMC1/CD44, COL4A2/CD44, and APP/CD74 ligand pairs dominate cellular communication in psoriasis. In contrast, the dominant ligand pairs in normal skin are DSG1/DSC3 and HBEGF/EGFR. Therefore, drug targeting of these ligand receptors holds potential clinical benefit in the treatment of psoriasis. The Pharos pharmacogenomic database identified six drugs targeting APP that are already in clinical use: tacrine, methylthioninium chloride, omapatrilat, Florbetapir F-18, Flutemetamol (18F), and florbetaben F18 (Figure 5).

### The distribution of cell type-specific regulons (CTSRs) within the intracellular regulatory network and their respective cell subtypes lends support to the hair follicle central axis hypothesis

In addition to exploring external signaling between cells, we delved into the role of the intracellular regulatory network in the central hair follicle-resident immune cell axis by analyzing cell type-specific regulons (CTSRs) (Supplementary Tables 2, 3). Quantitatively, B cells and T cells exhibited zero CTSRs, whereas resident immune cells around the hair follicle, such as keratinocytes, endothelial cells, and fibroblasts, demonstrated active CTSRs. Notably, the abnormal proliferation and differentiation of keratinocytes, a common feature of psoriasis, were validated by the presence of a substantial number of CTSRs (32 CTSRs, Supplementary Tables 2, 3), emphasizing their vital role in maintaining the regulatory network over time [41]. Endothelial cells and fibroblasts, known for their active participation in sustaining the central axis of the hair follicle [42], displayed robust CTSRs. Endothelial cells contributed to early angiogenesis in psoriasis, dependent on mediators like vascular endothelial growth factor, angiopoietin, and pro-angiogenic cytokines (23 CTSRs, Supplementary Tables 2, 3) [42]. Fibroblasts in the skin of psoriasis patients produced a variety of cytokines responsible for the chemical attraction of immune cells proliferating in the dermis and epidermis (16 CTSRs, Supplementary Tables 2, 3) [43].

Particularly noteworthy is the detection of STAT1 (Signal Transducer and Activator of Transcription 1), ZFX (Zinc Finger Protein X-Linked), IRF3 (Interferon Regulatory Factor 3), KLF5 (KLF Transcription Factor 5), NFIB (Nuclear Factor I B), and RXRA (Retinoid X Receptor Alpha) among the regulons during protein analysis of pathological sections. Their presence, upregulated compared to normal skin samples (Supplementary Tables 4, 5) [44], aligns with previous reports. For instance, STAT1, a transcription hub, inhibits the expression of metalloproteinases-3, influencing the cell adhesion molecule pathway and blood vessel formation [44]. STAT1 also affects the activity of the IL-22 promoter, which plays an important role in tissue repair and inflammatory responses [45, 46]. ZFX acts as an important regulator in the proliferation and migration of keratinocytes in skin wound healing. The miR-93-3p/ZFP36L1 (ZFP36 Ring Finger Protein Like 1) /ZFX axis promotes keratinocyte proliferation and migration, thereby affecting skin wound healing [47]. IRF3, a member of the IRF family, is implicated in the STING-IRF3 pathway, suggesting potential improvements in psoriasis treatment through its inhibition [48]. KLF5, a member of the Krupp-like factor subfamily, regulates sphingolipid metabolism and barrier function in the skin [49].

A significant finding supporting the hair follicle-immune cell axis in psoriasis is the involvement of the transcription factor NFIB in the maintenance and regeneration of hair follicles. Knockdown of NFIB and NFIX (Nuclear Factor I X) genes in mice results in the loss of epigenetic characteristics of hair follicle stem cells [50]. Our results indicate the presence of the transcription factor NFIB in keratinocytes, suggesting that the internal regulatory network of keratinocytes influences their interaction with hair follicles during immune regulation [50]. The pathological sectioning assay in our study revealed a substantial overlap exceeding 80% between the identified proteins and the target genes of CTSRs. This not only attests to the robustness of our results but also provides additional evidence of the intricate regulatory network operating within the cell. Specifically, the active factors associated with CTSRs are predominantly located in hair follicles and neighboring cells, while CTSRs such as B cells and T cells remain inactive (Supplementary Tables 2, 9, 10), aligning with the external communication patterns of the cell. This concurrence underscores the hair follicle central axis hypothesis from both the internal regulatory network and external information exchange patterns.

## DISCUSSION

Psoriasis, a multifaceted, chronic inflammatory skin condition involving diverse cell types, prompted our overarching objective: to unravel the communication patterns among these cell subpopulations, particularly focusing on their key ligand-receptor relationships at the single-cell level. This scrutiny aims to enhance therapeutic approaches through targeted interventions. Employing diverse analytical methods such as social network analysis, pattern recognition, and manifold learning techniques [28–31], we visually represented cell communication in this study to substantiate the central axis of hair follicles. Additionally, we leveraged pharmacogenomic information to identify cell type-specific regulons within the internal regulatory network, aligning with the external communication patterns, and identifying potential therapeutic targets [32]. Moreover, we utilized cutting-edge technology to discern proteins in limited diseased tissues, establishing a correlation between cellular communication patterns in psoriasis and their clinical implications. Furthermore, we confirmed the validity of the hair follicle central axis theory using pathological specimens.

### Immunosynergistic patterns of multiple immune cells in psoriasis

Previous investigations indicate an elevation of transitional B-cells in the peripheral blood of psoriasis patients compared to normal subjects, potentially as a compensatory mechanism triggered by inflammation [51–53]. Nevertheless, our data unveils a synchronization of immune cell communication in psoriasis, activating B-cells into an input signaling pattern that synergistically reinforces T-cell input signaling. In contrast, T-cells in normal skin exhibit a signaling output pattern, with B-cell signaling in an inactive state, underscoring the pivotal role of B-cells in psoriasis. This marks the inaugural report of a synergistic communication relationship between psoriatic B-cells and T-cells at the single-cell level.

Langerhans cells function as integral components of the dendritic cell immune regulatory network [54–56]. In response to antigenic stimulation, Langerhans cells migrate to locally draining lymph nodes, presenting antigenic peptides to T-cells, thereby initiating an antigen-specific immune response [54–56]. Our results unequivocally demonstrate that Langerhans cells dominate the output signaling pattern in psoriasis, primarily through the COLLAGEN and LAMININ signaling pathways. Beyond immune cell synergy, we identified hair follicle cells as mediators of communication among various cell types in psoriasis through the APP ligand. APP, a hallmark of skin aging, may contribute to the development of psoriatic inflammation [57].

### Shutdown of active signaling pathways in psoriasis

The cell communication patterns elucidated in this study reveal substantial suppression of normal T-cell signaling pathways (COLLAGEN, LAMININ, FN1, MPZ, and CD46) in psoriatic skin. Notably, only CD99 signaling remains consistently active, accompanied by MIF signaling. This suggests that CD99 serves as the foundational signal maintaining skin integrity, while MIF signaling emerges as a pivotal immune activation signal. In contrast, B-cells, primarily utilizing CD99, are prevalent in psoriasis, indicating a convergence of signaling pathways between these two immune cell types, potentially facilitating effective transmission and feedback of immune signals.

Of particular interest, psoriasis demonstrates the suppression of THBS, PERIOSTIN, MPZ, CDH1, NOTCH, CD46, CDH, and PDGF signaling pathways. THBS signaling’s impact on skin angiogenesis [58, 59], PERIOSTIN’s potential role in skin barrier dysfunction [58], and NOTCH signaling’s significance in the renewal of differentiated homeostasis in skin cells, with reduced expression linked to atopic dermatitis [39], underscore the intricate regulation occurring in psoriatic skin. Overall, the observed communication patterns among cells lean towards maintaining pathways that promote inflammation, such as APP and MIF, while actively inhibiting those associated with inflammation reduction.

### The hair follicle-psoriasis axis and its potential for therapeutic targeting

Abundant evidence substantiates the hair follicle-psoriasis hypothesis. Scalp psoriasis, for instance, correlates with sebaceous gland atrophy [60, 61]. The outer root sheath of the hair follicle houses melanocytes, Langerhans cells, and maintains close associations with resident immune cells such as keratinocytes, lymphatic endothelial cells, and mast cells, crucial for immune recruitment and activation [62–65]. The proximity of Langerhans cells, primary antigen-presenting cells in the epidermis [66], favors the hair follicle’s role in activating the local immune ‘garrison’. Our single-cell level analysis confirms the intricate communication of hair follicle cells with various cell types. Notably, our data aligns with the “hair follicle immune privilege” hypothesis, revealing no communication between hair follicle cells and effector T-cells [62, 65, 67, 68]. Instead, resident immune cells surrounding the hair follicle appear to play a pivotal role in immune regulation. Moreover, the communication patterns indicate a shift in the signaling pattern of psoriatic T-cells from an output to an input mode, potentially influenced by signals received from resident immune cells associated with the hair follicle.

Conventional anti-psoriatic drugs (e.g., dithranol, glucocorticoids, and cyclosporine A) necessitate uptake by skin cells to exert pharmacological effects. However, our study suggests an avenue for enhancing psoriasis treatment by inhibiting intercellular signaling pathways conducive to inflammation, such as APP. Six drugs targeting APP, already in clinical use for other diseases, were identified. Notably, Methylthioninium chloride, employed for years in treating dermatological conditions, particularly fungal inflammation [69, 70], emerged as a potential candidate. Our study of cell communication patterns advocates for clinical trials to rigorously assess the efficacy of these drugs for psoriasis treatment.

In conclusion, social network analysis, pattern recognition, and manifold learning techniques provided insights into communication patterns among diverse cell subtypes in psoriasis. Compared to normal skin, numerous signaling pathways are deactivated in psoriatic skin. Our study unveils the molecular characteristics of the hair follicle-psoriasis axis at the single-cell communication level, highlighting the dominance of resident immune cells in immune regulation and supporting the “immune privilege” hypothesis. Future developments in spatial technologies, including spatial proteomics and spatial single-cell transcriptomes in psoriatic inflammation, promise a more comprehensive understanding of spatially distinct inflammatory cell communication patterns [71–75].

## MATERIALS AND METHODS

### Single-cell communication pattern derivation

CellChat was utilized to read normalized average scRNA-seq expression levels for psoriatic and normal skin, and to merge cell type information to build the CellChat object format [28]. The ideal number of ligand-receptor pairings was calculated with CellChat’s more robust statistical approach “trimean” computeAveExpr which examines the expression levels of particular genes in a reciprocal manner: computeAveExpr(cellchat, features = c(“APP”), type = “truncatedMean”, trim = 0.1). A cellular communication network was developed using the computeCommunProb function. The likelihood of communication at the pathway level was computed using the computeCommunProbPathway function. The GEO (Gene Expression Omnibus database) dataset (GSE150672) was used to obtain raw single cell transcriptome data for psoriasis and normal skin.

### Quantifying single-cell communication patterns

Default settings for parameters cellchat-netAnalysis computeCentrality(cellchat, slot.name = “netP”) and netAnalysis signalingRole network (cellchat, signaling = pathways.show, width = 8, height = 2.5, font.size = 10) were employed to initially identify dominant senders, receivers, mediators, and influencers involved in inter-cell communication. Coordination among several signaling channels was examined concurrently, for which selectK(cellchat, pattern = “outgoing”) and selectK(cellchat, pattern = “incoming”) were used to obtain the number of communication patterns. The identifyCommunicationPatterns and netAnalysis river or netAnalysis dot functions were utilized to assign particular routes and illustrate these probable signaling patterns. Signal pathways were grouped together, and clusters were shown using the computeNetSimilarity, netEmbedding, netClustering, and netVisual embedding functions.

### Differential examination of the communication patterns of a single cell

The total number and strength of interactions were compared with the compareInteractions function. xpd = TRUE (cellchat, weight.scale = T), mfrow = c (1,2) netVisual diffInteraction netVisual_ diffInteraction (weight.scale = T, “weight” as measure). The netVisual heatmap function displays the number and intensity of cell interactions as a heat map. The netAnalysis signalingRole scatter function was utilized to examine the various functions of cell populations, such as signal transmission and reception. The netVisual bubble function makes it feasible to identify malfunctioning signaling.

### Analysis of cell type-specific regulons (CTSRs) in distinct psoriasis cell subtypes

Single-cell gene expression profiles for psoriasis were organized into Seurat objects. To ensure downstream analysis reliability, genes with zero expression values in >99.9% of cells and cells with fewer than 200 genes expressing zero values were excluded. Subsequently, the bicluster tool QUBIC2 was employed for gene module delineation. Biclusters, representing co-expressed genes in specific cell subpopulations, were identified [76]. The similarity between cells in the cell type cluster and those in the bicluster was examined. The hypothesis was that genes constituting the bicluster would exhibit a regulatory signaling response in specific cell types. Hypergeometric enrichment tests were conducted to assess the consistency of cell types in the dataset and the cell composition of the bicluster. A significant result (adjusted P < 0.05) in cellular hypergeometric tests indicated the activation of the bicluster in the respective cell type. Active gene modules with cell type specificity and motifs were identified in the biclusters. Anticipated functions were determined using MEME and DMINDA2.0 [77, 78], and de novo motif development was performed. Upstream promoter sequences within a 2,000 bp region were selected, using the hg38 reference genome. The R package BSgenome.Hsapiens.UCSC.hg38 provided the hg38 human reference genomes. Motif clustering in distinct cell types was analyzed using TOMTOM [79] and the most similar known motifs were annotated from the HOCOMOCO database [80]. To filter out matching motifs, HOCOMOCO targets with a Q-value larger than 0.05 were excluded. The Q-value represented the false discovery rate at which the observed similarity was considered significant. For each theme cluster, the list of non-redundant genes was designated as a regulon. The regulon activity score (RAS) in the cell was generated based on the ranking of expression levels of all involved genes. An empirical P-value, calculated by comparing the RSS (Regulon Specificity Score) of a regulon against a randomly selected set of genes from the same cell type (by bootstrap method), led to the identification of cell-specific regulons with a P-value of 0.05 or less. The single-cell dataset of mouse skin at various developmental phases was derived from GSE129218.

### Analysis of medication targeting based on ligand-receptor combinations on a single cell level

Several ligand-receptor combinations were entered into the Pharos database, and the “bulk search” function was used to find potential psoriasis therapies [81]. Choices were divided into four basic categories: “Tclin” (drugs having a known mechanism of action and FDA approval), “Tchem” (drugs with a small molecule ligand linked to the target protein), “Tbio” (known gene ontology or phenotype), and “Tdark” (not yet studied). Due to its immediate therapeutic significance, the “Tclin” class of medicines was chosen for future investigation.

### Clinicopathological samples

A total of 225 clinical pathological samples were collected for proteomic sequencing, of which 135 came from 9 patients with psoriasis and 90 from 6 individuals with normal skin. Supplementary Table 1 provides a summary of the clinicopathological characteristics of all patients included in this study. Prior informed consent was obtained, and the Shenzhen Maternity & Child Healthcare Hospital’s Ethics Committee authorized this study (SFYLS [2022] 044).

### Mass spectrometric (MS) analysis preparation for formalin-fixed, paraffin-embedded (FFPE) tissue

Psoriatic skin sections and paraffin sections from patients undergoing debulking surgery were collected by pathologists at the Shenzhen Maternal and Child Healthcare Hospital. The stored samples at -80° C were retrieved, dewaxed, and diluted fourfold with lysis buffer (1% Sodium dodecyl sulfate (SDC) containing 1% protease inhibitor). Subsequently, they were decrosslinked overnight at 56° C, lysed through ultrasonication, and centrifuged at 12,000 g for 10 minutes. Following centrifugation, removal of cell debris was performed, and the supernatant was carefully transferred to new centrifuge tubes. From each sample, 5 μl of supernatant was extracted for silver staining.

DL-Dithiothreitol (DTT) was introduced to adjust the sample concentration to 5 mM. The samples were then heated at 56° C for 30 minutes after adjusting the volume to match that of the lysate, ensuring equal protein quantities for each sample. Iodoacetamide (IAM) was added to adjust the sample concentration to 11 mM before an incubation at room temperature in the dark for 15 minutes. Urea was diluted with triethylammonium bicarbonate buffer (TEAB) to achieve a concentration of less than 2 M. Trypsin was added to the samples at a ratio of 1:50 (m/m) to protein, thoroughly mixed, and subjected to overnight digestion. An additional 4 hours of digestion were carried out by introducing trypsin to the samples at a ratio of 1:100 (m/m) to protein.

### Liquid chromatography-mass spectrometric evaluation of FFPE tissue samples

The peptides were solubilized in mobile phase A and separated using an EASY-nLC 1200 ultra-high-performance liquid chromatography system (LC140, Thermo ScientificTM, Waltham, MA, USA). Mobile phase A comprised an aqueous solution containing 0.1% formic acid and 2% acetonitrile, while mobile phase B consisted of an aqueous solution containing 0.1% formic acid and 90% acetonitrile. The gradient parameters were as follows: 0-70 min, 6%-22% B; 70.0-83.0 min, 22%-34% B; 83.0-87.0 min, 34%-80% B; 87.0-90.0 min, 80% B. After separation, the peptides entered a Nanospray Flex (NSI) ion source (Nanospray FlexTM, Thermo ScientificTM, Waltham, MA, USA) and underwent analysis using a high-resolution Orbitrap ExplorisTM 480 mass spectrometer (Thermo ScientificTM, Waltham, MA, USA).

The ion source voltage was set at 2.2 kV, and the FAIMS compensation voltage (CV) varied between -45 V and -65 V. Peptide parent ions and their secondary fragments were identified and analyzed by mass spectrometry across the scan range 400 - 1200 m/z, with a resolution set at 60,000. The secondary mass spectrometry scan range was 110 m/z, and the secondary scan resolution was 15,000, with TurboTMT turned off. After the initial scan, the 25 peptide ions with the highest signal intensity were selected using a data-dependent acquisition (DDA) approach. Employing a fragmentation energy of 28%, the peptide parent ions were successively fragmented using the higher-energy collisional dissociation (HCD) cell for secondary mass spectrometry. To enhance data presentation in the output spectra, the automatic gain control (AGC) was set to 100%, the signal threshold was 5.0e4 ions/s, the maximum injection time was set to Auto, and the dynamic exclusion time for tandem mass spectrometry scans was 25 seconds to prevent repeated scanning of the parent ions.

### Analysis of unprocessed proteomics data

The raw mass spectrometry data were imported into the library search software, and analytical parameters were configured according to the experimental protocol. The secondary mass spectrometry data were scrutinized using Proteome Discoverer (v2.4.1.15) [38]. Search parameters included Homo_sapiens_9606_SP_20220107.fasta (20376 sequences). An inverse library was incorporated to calculate the false discovery rate (FDR) due to random matching, and a common contamination library was added to the database to eliminate contaminating proteins. The digestion method was specified as Trypsin (Full), with the number of missed sites set to 2. The minimum peptide length was established at 6 amino acid residues, and the maximum number of peptide modifications allowed was 3. The mass error tolerance for primary parent ions was set at 10 ppm, and for secondary fragment ions, it was set at 0.02 Da. Carbamidomethyl (C) was designated as a fixed modification, while Oxidation (M), Acetyl (N-terminus), Met-loss (M), and Met-loss+acetyl (M) were considered variable modifications. The FDR for protein, peptide, and peptide-spectrum matches (PSM) identification was maintained at 1%. To ensure high-quality analysis, additional data filtering was applied to the library search results, requiring identified proteins to contain at least one specific (unique) peptide.

### Diversification of the protein’s functional properties

Gene Ontology (GO) proteome annotations were extracted from the UniProt-GO database (http://www.ebi.ac.uk/GOA/) [82]. Protein IDs were converted to UniProt IDs and mapped to GO IDs, employing InterProScan and protein sequence-matching algorithms to extend GO functional annotations to newly discovered proteins not annotated by the UniProt-GO database [83]. The proteins were categorized into biological processes, cellular components, and molecular activities based on Gene Ontology annotations. Enrichment analysis for differentially expressed proteins against all identified proteins was conducted using a two-tailed Fisher’s exact test. A corrected GO with a p-value below 0.05 was deemed significant.

Enriched pathways were identified using the Kyoto Encyclopedia of Genes and Genomes (KEGG) database. The degree to which differentially expressed proteins were enriched for all discovered proteins was assessed through a two-tailed Fisher’s exact test [84]. Significant pathways, determined by a corrected p-value of 0.05 or below, were hierarchically organized according to the KEGG website.

Hierarchical clustering based on the functional classification of differentially expressed proteins (e.g., GOs, domains, pathways, and complexes) was performed. Categories with P-values less than 0.05 enriched in at least one cluster were retained. A filtered P-value matrix was generated using the x = -log10 formula (P-value), and a z-transformation was applied to these x-values for each functional category. The resulting Z-values were clustered in Genesis using one-way hierarchical clustering (Euclidean distance, mean linkage clustering). Clustering associations were visualized using the “heatmap.2” function of the “gplots” or “pheatmap” R packages.

Functional analysis of proteins, including classification into families and prediction of structural domains and significant sites, was conducted using the InterPro database (https://www.ebi.ac.uk/interpro). A two-tailed Fisher exact test assessed the enrichment of differentially expressed proteins for all identified proteins [85], with significance accepted at a p-value less than 0.05 after correction. Enrichment of functional structural domains in differentially expressed proteins was explored using the Pfam database [86], with significance determined by Fisher’s exact test at a p-value less than 0.05.

The EggNOG database, known for annotating clusters of homologous proteins or COGs (Clusters of Orthologous Groups) [87], provided comprehensive taxonomic information, homologous protein sequences, phylogenetic tree construction, and functional annotation for each homologous gene cluster, surpassing current databases like the NCBI COG database.

### Protein subcellular localization

To predict subcellular localization, we employed WolfPsort, a tool for subcellular localization prediction. WoLF PSORT is an enhanced version of PSORT/PSORT II specifically designed for predicting eukaryotic sequences [88, 89].

## Supplementary Material

Supplementary Figures

Supplementary Table 1

Supplementary Table 2

Supplementary Table 3

Supplementary Table 4

Supplementary Table 5

Supplementary Table 6

Supplementary Table 7

Supplementary Table 8

Supplementary Table 9

Supplementary Tables 10-14
