# Supplementary material for: Generating detailed intercellular communication patterns in psoriasis at the single-cell level using social networking, pattern recognition, and manifold learning methods to optimize treatment strategies
Source: Aging (Albany NY). 2024 Jan 29;16(3):2194–231. doi: 10.18632/aging.205478 (PMC10911347; doi:10.18632/aging.205478)
Supplement: Supplementary Figures [file aging-16-205478-s001.pdf]

SUPPLEMENTARY FIGURES

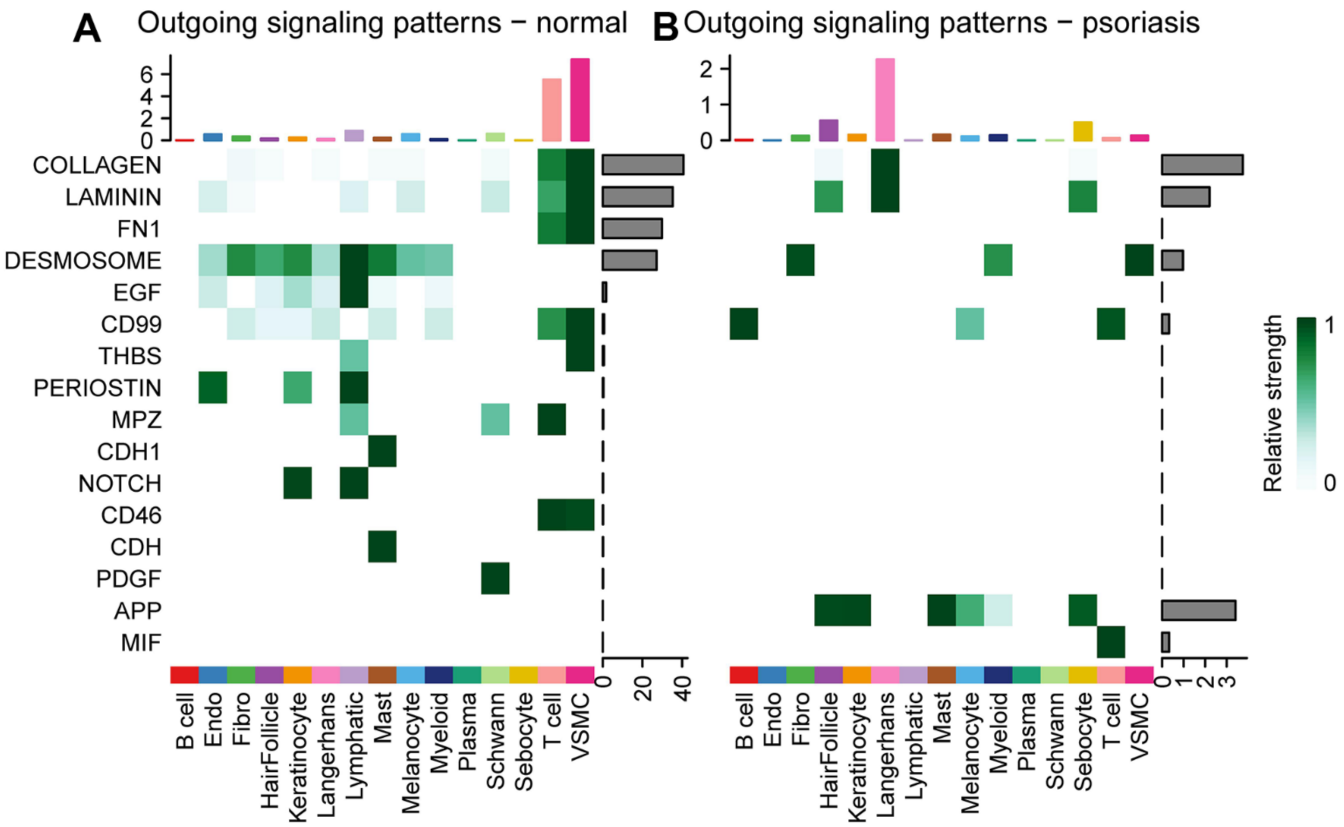

Supplementary Figure 1. Outgoing signaling patterns for each subtype of cells in psoriatic and normal skin. (**A**) Normal skin. (**B**) Psoriasis.

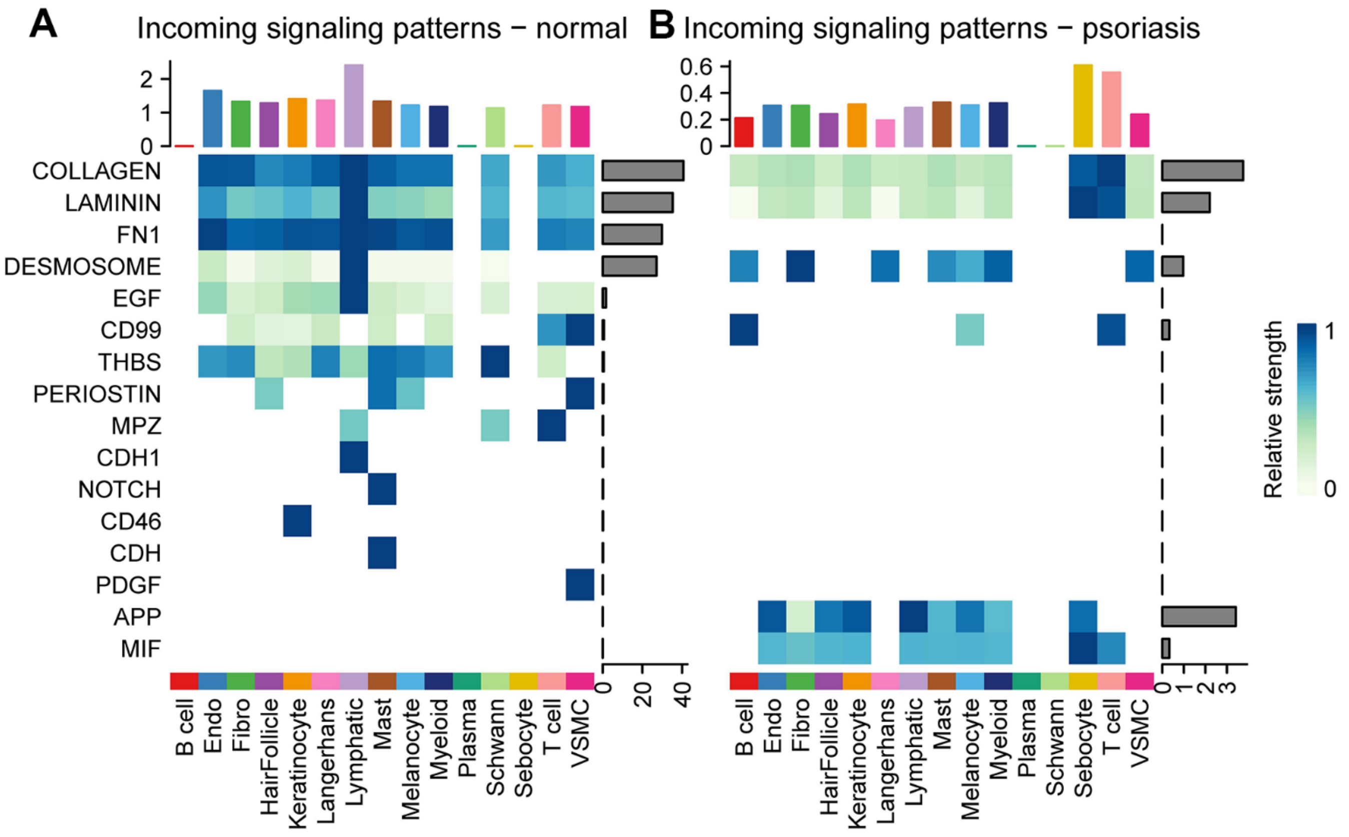

Supplementary Figure 2. Incoming signaling patterns for each subtype of cells in psoriatic and normal skin. (**A**) Normal skin. (**B**) Psoriasis.

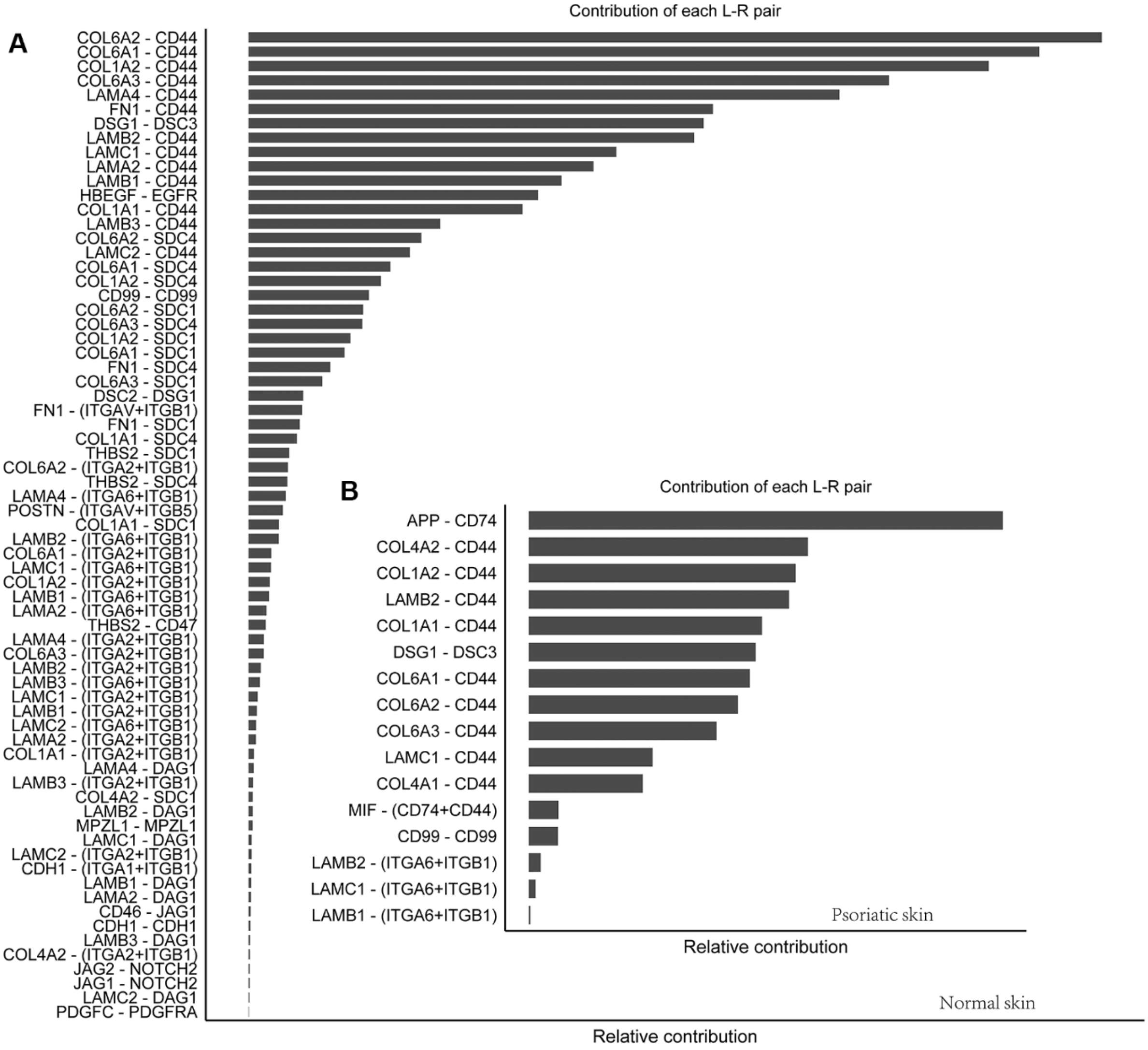

C

Detection status	Proportion	Ligand receptor
Detectable proteins in pathology sections	36 (36/80, 45%)	COL1A1, COL1A2, COL4A1, COL4A2, COL6A1, COL6A2, COL6A3, LAMA4, LAMA5, LAMB1, LAMB2, LAMC1, APP, DSC1, DSG1, NAMPT, MIF, ANGPTL2, THBS1, HSPG2, CD99, GRN, SELPLG, ITGA1, CD44, SDC1, ITGA6, DAG1, CD74, DSC3, NCL, CDH5, CD47, SORT1, ITGB1, ITGB4
Unidentified protein at the pathology section	44 (44/80, 55%)	MDK, TNFSF10, ANGPTL4, DLL1, DLL4, JAG2, VEGFB, PGF, APLN, SELE, TNF, MPZL1, ADM, EFNA1, EFNA5, SEMA6A, ICOSL, EFNB1, EFNB2, IGF2, SEMA7A, EDN1, TNFSF12, ITGA9, ITGA10, SDC4, INSR, ACKR3, TNFRSF10B, TLR4, NOTCH1, NOTCH4, VEGFR1, APLNR, TNFRSF1B, CALCRL, EPHA1, EPHA4, PLXNA2, ICOS, CTLA4, EDNRB, TNFRSF12A, CXCR2

Supplementary Figure 3. Contribution of ligand receptors (L-R) in normal skin and psoriatic skin. (**A**) Contribution of ligand-receptor pairs in normal skin. (**B**) Contribution of ligand-receptor pairs in psoriatic skin. (**C**) Expression of ligand receptor pairs in pathological tissue sections and the proportion of them detected. The data show that all ligand receptor pairs have been detected in psoriasis and that a relatively high proportion of ligand receptors in normal skin have also been detected for the most part.

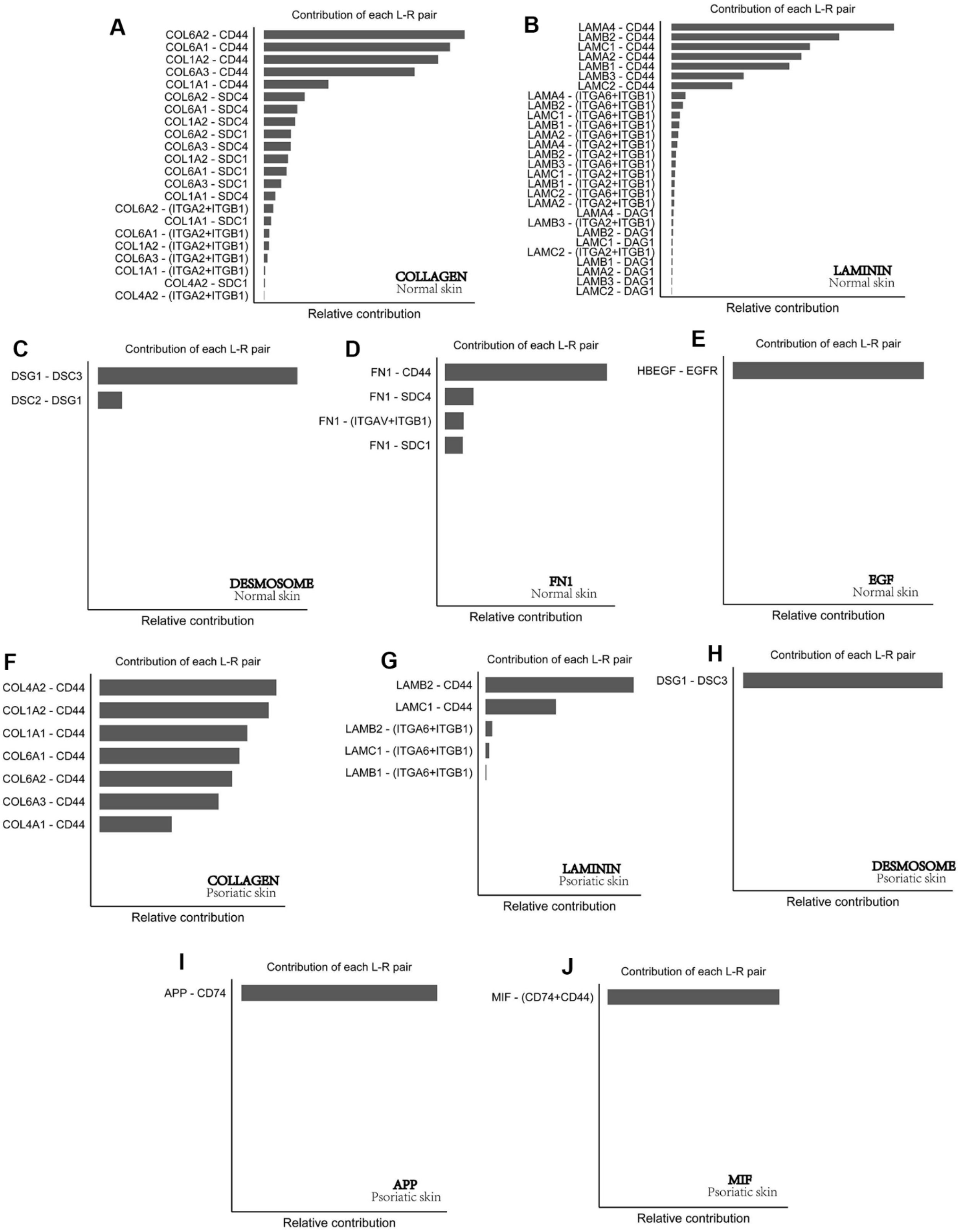

Supplementary Figure 4. Contribution of ligand receptor (L-R) in different signaling pathways in psoriasis and normal skin. (**A**–**E**) Contribution of ligand receptor pairs in five signaling pathways (COLLAGEN, LAMININ, FN1, DESMOSOME and EGF) in normal skin. (**F**–**J**) Contribution of ligand receptor pairs in five signaling pathways (COLLAGEN, LAMININ, DESMOSOME, APP and MIF) in psoriatic skin.
