# Supplementary material for: Generating detailed intercellular communication patterns in psoriasis at the single-cell level using social networking, pattern recognition, and manifold learning methods to optimize treatment strategies
Source: Aging (Albany NY). 2024 Jan 29;16(3):2194–231. doi: 10.18632/aging.205478 (PMC10911347; doi:10.18632/aging.205478)
Supplement: Supplementary Table 2 [file aging-16-205478-s003.pdf]

Supplementary Table 2. The cell type-specific regulons (CTSRs) in psoriasis.

Num.	Cell clusters	Number of regulons	Number of CTSRs	CTSRs
1	B cell	0	0	-
2	Endo	23	23	E2F7, ETS2, IRF3, TBX21, NFAC1, ZN467, SP2, VEZF1, SPIB, FLI1, KLF5, PRDM6, MAZ, ZN341, PTF1A, STAT1, PATZ1, ZN263, SP3, SP1, E2F6, WT1, CPEB1
3	Fibro	18	16	SP4, ZN350, LMX1A, TFE2, VEZF1, PATZ1, KLF12, ZN467, TBX1, ZN263, RXRA, CPEB1, SP1, ZN770, Z324A, SP3
4	Hair Follicle	5	2	VEZF1, ZNF770
5	Keratinocyte	32	32	SALL4, IRF3, ZN341, PBX1, TBX1, ZN467, COT1, NFIB, NFAC1, ZN263, SRBP2, EGR4, ETS2, TBX15, PRDM6, SPIB, RARB, KLF3, RXRA, WT1, FOXJ3, SP3, CPEB1, KLF1, PATZ1, MAZ, SP1, ZFX, SP2, ZN770, PITX2, VEZF1
6	Langerhans	0	0	-
7	Lymphatic	1	0	-
8	Mast	2	1	ZNF148
9	Melanocyte	0	0	-
10	Myeloid	3	0	-
11	Plasma	17	0	-
12	Schwann	16	0	-
13	Sebocyte	4	0	-
14	T cell	2	0	-
15	VSMC	7	2	CPEB1, SP2
