# Supplementary material for: Generating detailed intercellular communication patterns in psoriasis at the single-cell level using social networking, pattern recognition, and manifold learning methods to optimize treatment strategies
Source: Aging (Albany NY). 2024 Jan 29;16(3):2194–231. doi: 10.18632/aging.205478 (PMC10911347; doi:10.18632/aging.205478)
Supplement: Supplementary Table 4 [file aging-16-205478-s005.pdf]

**Supplementary Table 4. Human and mouse skin share overlapping cell type specific regulons (CTSRs).**

| Dataset | Overlapping regulons | All regulons | Method |
|---|---|---|---|
| Human skin in psoriasis | ETS2, IRF3, SP2, FLI1, KLF5, MAZ, STAT1, SP1, WT1, SP4, SALL4, SRBP2, FOXJ3, PITX2, ZN148, ZN281 | ETS2, IRF3, SP2, FLI1, KLF5, MAZ, STAT1, SP1, WT1, SP4, SALL4, SRBP2, FOXJ3, PITX2, ZN148, ZN281, E2F7, TBX21, NFAC1, ZN467, VEZF1, SPIB, PRDM6, ZN341, PTF1A, PATZ1, ZN263, SP3, E2F6, CPEB1, ZN350, LMX1A, TFE2, KLF12, TBX1, RXRA, ZN770, Z324A, PBX1, COT1, NFIB, EGR4, TBX15, RARB, KLF3, KLF1, ZFX, KAISO, HXD13, RARA | IRIS3 |
| Mouse skin cell (anagen, 5w) | ETS2, IRF3, SP2, FLI1, KLF5, MAZ, STAT1, SP1, WT1, SP4, SALL4, SRBP2, FOXJ3, PITX2, ZN281 | ETS2, IRF3, SP2, SPIB, FLI1, KLF5, MAZ, STAT1, SP3, SP1, WT1, SP4, SALL4, SRBP2, KLF3, FOXJ3, ZFX, PITX2, ZN281, HMGA1, SPI1, STF1, ERR2, SALL1, ZN143, FUBP1, ETV2, NR5A2, BATF, EGR1, SRY, ELF5, FOXJ2, KLF15, ANDR, SP5, HNF6, BHA15, RXRG, SMAD4, RFX6, THA, KLF6, ZBT17, HAND1, FOXD3, STA5A, MYB, THA11, IRF4, ZN322, FOXQ1, RREB1, FOXD1, RUNX3, E2F1, MAFK | IRIS3 |
| Mouse skin cell (telogen, 9w) | ETS2, IRF3, SP2, FLI1, KLF5, MAZ, STAT1, SP1, WT1, SP4, SALL4, SRBP2, FOXJ3, PITX2, ZN148, ZN281 | ETS2, IRF3, SP2, FLI1, KLF5, MAZ, STAT1, SP1, WT1, SP4, SALL4, SRBP2, FOXJ3, PITX2, ZN148, ZN281, ESR1, RREB1, PRD16, OLIG2, STF1, GLI3, FUBP1, FOXJ2, SALL1, SP5, NR2C1, ELF5, PURA, EGR2, ANDR, ETV2, SOX9, SMAD3, ZBT17, SMAD2, KLF15, E2F3, IRF5, SMAD4, NFIC, RXRG, SRY, SPI1, ERR2 | IRIS3 |
