# Supplementary material for: Generating detailed intercellular communication patterns in psoriasis at the single-cell level using social networking, pattern recognition, and manifold learning methods to optimize treatment strategies
Source: Aging (Albany NY). 2024 Jan 29;16(3):2194–231. doi: 10.18632/aging.205478 (PMC10911347; doi:10.18632/aging.205478)
Supplement: Supplementary Table 6 [file aging-16-205478-s007.pdf]

Supplementary Table 6. Overlapping ligand receptors in protein assays in pathological sections of psoriasis and healthy skin.

Protein accession	Gene name
Q9UKU9	ANGPTL2
P05067	APP
P16070	CD44
Q08722	CD47
P04233	CD74
P14209	CD99
P33151	CDH5
P02452	COL1A1
P08123	COL1A2
P02462	COL4A1
P08572	COL4A2
P12109	COL6A1
P12110	COL6A2
P12111	COL6A3
Q14118	DAG1
Q08554	DSC1
Q14574	DSC3
Q02413	DSG1
P28799	GRN
P98160	HSPG2
P56199	ITGA1
P23229	ITGA6
P05556	ITGB1
P16144	ITGB4
Q16363	LAMA4
O15230	LAMA5
P07942	LAMB1
P55268	LAMB2
P11047	LAMC1
P14174	MIF
P43490	NAMPT
P19338	NCL
P18827	SDC1
Q14242	SELPLG
Q99523	SORT1
P07996	THBS1
