# Supplementary material for: Generating detailed intercellular communication patterns in psoriasis at the single-cell level using social networking, pattern recognition, and manifold learning methods to optimize treatment strategies
Source: Aging (Albany NY). 2024 Jan 29;16(3):2194–231. doi: 10.18632/aging.205478 (PMC10911347; doi:10.18632/aging.205478)

Supplementary Table 10. Proportion of target genes for cell type specific regulators (CTSRs) detected in psoriasis and healthy skin pathology tissue sections.

Detection status	proportion	Target genes of cell type specific regulators (CTSRs)
Detectable proteins in pathology sections	674 (674/839, 80.3%)	TPM4, WDR1, RPL29, TPM1, TMSB4X, UBQLN1, YME1L1, PTBP3, RPL23, CCT3, PTPRF, PTBP1, RPS27A, SMARCC1, TPR, OSBPL8, RAB31, RPS12, SKP1, SUCLG1, PSMD1, RAC1, RPL8, RPL36, PSMD2, RPL12, RPL10A, RPL10, SH3BGRL3, YY1, UBE2K, TXN, PTMA, PUF60, RAB10, RSL1D1, RPL34, SEC62, USP15, NAA50, CYB5R3, PPIG, RPL27A, RPL28, VAMP8, RIOK3, RPL37A, RPL5, AHCY, SYNGR2, SPTBN1, VPS26A, VIM, PLS3, RPN2, HNRNPAB, SNRPD2, SFPQ, SLPI, RPL32, SERF2, RPS14, S100A14, SUB1, TCP1, TGOLN2, CCT2, STAT1, RANGAP1, RAD23B, PRNP, SBSN, SOD2, SET, TXNDC17, TYMP, PSMB7, RPL26, RPL4, ROCK2, UTRN, VWF, XRCC6, RPL6, TPM3, ATP6V1F, ATP6V1G1, OS9, PSMA6, RPL30, RPLP2, UQCR10, SON, RAB34, REEP5, RHOA, RHOC, RPL14, RPL13A, RPL39, POLR2L, POLR1D, MRPL12, SSR4, CCT8, XRCC5, RRBP1, RPS19, RPS16, RPS28, SRP72, SRP14, TERF2IP, NDUFS5, TM9SF3, PSMD7, RPL24, RPL31, TPSAB1, RPLP0, TRIM29, RPS13, RPS15A, YBX3, RPS21, S100A16, CCT5, TUBB2A, UBXN1, ZFP36, ZC3H15, UBXN4, TIMM13, TUBB4B, TXNIP, ZNF207, ZNF385A, VDAC1, RPS3, SLK, TIMP1, THY1, USP47, WNK1, PCOLCE, PLVAP, PSMB5, RPL36AL, SPARC, ZBTB20, HDLBP, RBM25, UBL5, WAC, ZFP91, YWHAB, YWHAQ, ACTG1, CBR1, CCDC80, CD93, ANGPTL2, COL5A1, COL6A3, BTF3, BAG1, BICD2, CYB5A, DCTN3, CAV1, CAPZB, DEK, DDX21, DDX3X, CFH, CD63, COX5A, EGFR, NT5E, HSPE1, AP2S1, ATP1A1, ANXA2, CTSG, PSIP1, CLTA, HSPA5, CALR, DAB2, CDH11, CHD4, COX7C, DPYSL3, EIF1, EEF2, IFI16, CAPRIN1, CKAP4, CNBP, COL1A2, EDF1, CTNNB1, G3BP1, DSTN, HADHA, ECHS1, GSN, BAIAP2, SLC25A5, CAPG, CPD, CPA3, ARPC5, COL1A1, CSDE1, TMBIM6, BRK1, CYBRD1, CALD1, CALU, COL4A1, GSPT1, FUS, FTL, DBI, GOT2, AKAP13, PYCARD, CRTAP, COPS6, COX5B, COX6C, BCAP31, BAG3, CAP1, C1S, COX7A2, A2M, ABLIM1, YWHAZ, CTSK, CD44, CLUH, CFL1, CSTA, GRN, HMGCS1, HMGA1, AHNAK, APRT, ACTR3, ARPC2, EPB41L2, DSC2, EBNA1BP2, DSC3, DYNC1H1, EIF3M, GPNMB, EEA1, CSTB, GGCT, ARHGDIA, ENO1, EIF3D, GUK1, PKM, HSPA9, ALDOA, CAPN1, ARL8B, ANP32B, ACTR2, ARPC5L, CNN3, COL18A1, CCAR1, DDX1, CST3, SFN, DEGS1, SDF4, CD99, EI24, CTNNA1, COX6B1, GBP2, FAM162A, ENSA, GSTP1, YWHAG, APP, ALDH2, ALDH3A2, AKR1B10, AQP3, ANXA1, ATP2A2, CCDC50, CHMP2A, CD9, COX4I1, CYCS, GNG12, GNG11, ITGA6, AEBP1, ANXA5, SYNCRIP, EIF5, EIF6, LRP1, MAN1A1, METAP1, MBD2, LAMC1, LAMP2, KTN1, MDH2, MAL2, NCL, MYL12B, CD59, CD81, CLTB, COL3A1, COL5A2, CLIC4, COL6A1, COL6A2, FBLN2, GLG1, HSPH1, HSPA4L, HNRNPF, PPA1, IQGAP1, IMPDH2, LYPD3, MTDH, LUM, MAPK13, CDC42, EZR, ILF2, EIF4A3, EIF5B, EIF4B, SERPING1, CD74, MAP7D1, LY6D, MGLL, MFAP4, FDPS, GHITM, EIF2S1, GNG5, HSP90AB1, HNRNPC, HNRNPU, LRRFIP1, IDH2, IDI1, MBNL1, EIF4A1, IGFBP7, LAMB1, KRTDAP, CLU, DSG1, DSG3, CLCA2, DDX17, EIF3B, EEF1A1, FGFBP1, PERP, NDUFB9, NDUFB10, NHP2, CALM1, CEBPD, CIRBP, DYNLL1, COL15A1, CYC1, CTR9, FLNA, ETF1, ESRP1, FSTL1, HSD17B10, HTRA1, MSN, DRAP1, NDUFB4, SLC25A6, GJA1, CDH1, DDX5, DSP, DIAPH1, ENAH, TUFM, FMOD, FBN1, FBLN1, LAMA4, RPS6KB2, FTH1, LGALS3, LPAR1, NFIX, NNMT, NUCKS1, NAA15, CALML3, CTSD, DNAJA1, EIF3J, EIF3I, ITM2B, PDAP1, CCDC47, ATP1B3, HSPD1, DHCR24, EIF3H, GLUL, GNA15, GNAI2, GLTP, SLC2A1, HEBP2, HNRNPD, KHDRBS1, HSPA8, IFITM3, EIF2S2, NFKBIA, HK1, MMP2, AK2, LAD1, NSA2, NPM1, OLFML3, OPTN, ENG, EIF3E, EIF3L, VCAN, DAD1, DNAJB1, EIF3A, HSP90B1, ITGA5, KRT1, SERBP1, PDLIM1, IGFBP4, MCL1, MATR3, EIF4G2, MARCKS, MX1, JUP, PPP1CA, PPP1CB, PSMA4, GPX4, LAMP1,

		HSP90AA1, KPNB1, JUNB, JUND, ITGB5, PARK7, PALLD, PLP2, HINT1, NDUFS7, NME1, NDRG1, NOLC1, NDUFA4, NDUFA10, MYL6, IMPA2, LGALS3BP, SLC16A10, PRDX1, PRDX5, NRP1, RBM3, P4HB, PDIA3, PDIA6, PSMC5, PSMB1, HSPA4, NACA, PA2G4, PGRMC1, PDGFRB, PEA15, UQCRQ, C6orf132, CHCHD2, CD82, GAPDH, HDGF, HNRNPDL, IL6ST, ITGB1, PSMC2, CSNK1A1, LGALS1, HNRNPK, PAFAH1B1, KLF5, MXRA5, NDUFV1, PSMB6, RBM39, GOLGA4, NDUFA13, PAPOLA, DCN, SFRP2, SLIT3, SNRPD3, TOLLIP, SRSF11, DNTTIP2, GLO1, MACF1, PFDN5, PABPC1, PRPF40A, RAD21, RAB1A, RAB25, RPL27, RPL38, S100A4, S100A6, S100A7, S100A8, S100A9, S100A10, S100A11, SAP18, SEC61B, PTP4A2, FAM114A1, RPL22, RPL23A, RPS23, RPS24, SNRPB, MRPS34, PKP3, PSMA3, PPIB, UQCRH, CRABP2, MRPL27, RPL13, PSMD8, HNRNPA2B1, RPS6, PTGES3, SNX9, SRGN, TPI1, RPL19, SF3B1, RPS15, CCT6A, TAF7, PCBP2, PKP1, SEC31A, TUBA4A, SRSF3, STK24, U2SURP, RPS5, TALDO1, TAGLN2, GM2A, SDC1, AHCYL1, NAPA, TMEM45A, TIMM8B, SPINT2, STAT3, SWAP70, GNB1, GDI2, CAST, EIF4G1, PSMA7, RAB3D, RPS29, HSPB1, MYH9, NCKAP1, HSPG2, PRRC2C, HNRNPM, NDUFS6, METAP2, MAP4, UQCRB, RAB7A, RPL18, RPLP1, RPL3, RRAGC, SOD1, SH3GLB1, RPS20, RPS11, SERPINB4, TMEM245, TMED3, PCBP1, PPL, PHB2, RPL11, PSMC3, RPL15, TUBA1C, SRSF2, TMED9, MZT2B, RPS8, RPS2, RPSA, TACC1, TACSTD2, VCP, SERPINB5, SPCS1, MSMO1, PEBP1, PGK1, NOP58, RPL35, NDUFB7, PMVK, PPP3CA, PLEKHO2, PLIN3, PLXND1, ARHGAP18, PRRX1, RPS7, FAU, RTN4, SYNE2, LTBP1, MPZL2, SLC25A3, RAN, PFN1, PSMB4, PPIA, RPL35A, STOM
Undetectable proteins at the pathology sections	165 (165/839, 19.7%)	EPAS1, TCF4, TGFBR2, TM4SF1, EMP1, NUAK1, EMCN, ETS2, SPARCL1, ATP8B1, EVA1C, HIPK3, OLFM1, CAV2, PIK3C2A, SNRK, TSC22D1, TNFRSF10D, ETS1, RALGAPA2, ATF4, CDKN1A, FOXP1, ITM2A, PRKCH, PTTG1IP, BMPR2, ECE1, FLT1, PRSS23, WWTR1, CCNI, IFITM2, JAK1, SH3BP5, ADAMTSL4-AS1, DUSP6, APLP2, ARID5B, ATF3, EID1, FOS, GADD45B, IER5, RHOB, RPL37, ZFP36L1, ZFP36L2, CRISPLD2, APCDD1, TIMP2, F2R, LAPTM4A, ACTB, FGFR1, IL1R1, PMP22, TMEM176B, DDR2, PAM, NUPR1, PLAC9, SERINC1, ANTXR1, ID2, REV3L, NR4A1, ARRDC3, AKAP9, ARF1, DMKN, FXYD3, SNHG5, TSPO, ZFAS1, C4orf3, KRT10, KRT6A, SCD, EMP2, SPTSSA, MAFB, CA12, KRT16, NRARP, NSG1, COX8A, DUSP1, EGR1, IER2, OST4, SLC39A6, TMEM123, RHOV, CALM2, GADD45GIP1, MAF, RALBP1, UBE2D3, EHF, GJB2, BHLHE40, COX6A1, COX7B, EFNA1, MAPK6, MRFAP1, RND3, SNHG8, CAMLG, NFE2L2, PTP4A1, RAB11A, TOMM20, C19orf33, C1orf21, KLF4, NDUFB2, PYURF, SERP1, BPTF, NFE2L1, ALDH3B2, C11orf58, C1orf43, MRPL51, NDUFA1, POMP, PPP2CA, ROMO1, SLC25A39, TOMM7, MYL12A, RORA, MXD1, SPPL3, ANKRD12, AURKAIP1, FOSB, GAS5, PNRC1, PPDPF, RNF11, RSRC2, SLC38A2, TMEM154, TNFAIP3, ZC3H12A, ZDHHC3, SCAF11, SCO2, BMP2K, KIT, LAPTM5, BTG1, MALAT1, OAZ1, JUN, PPP1R15A, UBA52, UBB, UBC, KLF6, SLU7, NFKBIZ

Supplementary Table 11. KEGG pathway enrichment analysis of Q1 protein in psoriasis pathology tissue sections.

KEGG pathway	Mapping	Background	All mapping	All background	Fold enrichment	Fisher's exact test p-value	-log10 (p-value)	Related proteins
hsa04610 Complement and coagulation cascades	21	64	149	2834	6.24	2.02707152308558E-12	11.69	P00747 P04004 P13671 P01024 P08697 P00742 P04003 P0C0L5 P02679 P03952 P10909 P02748 P07357 P02675 P08174 P02671 P00748 P05154 P05546 P01042 P00734
hsa05143 African trypanosomiasis	5	14	149	2834	6.79	0.000511397243201717	3.29	P69905 P02647 P50148 P68871 P01042
hsa03320 PPAR signaling pathway	7	38	149	2834	3.5	0.00306959200311731	2.51	Q9BX66 Q99541 P23786 P02647 P02652 O15540 O60240
hsa00860 Porphyrin metabolism	4	15	149	2834	5.07	0.00635633605691733	2.2	P00450 P13716 P30519 P30043
hsa04080 Neuroactive ligand-receptor interaction	4	16	149	2834	4.76	0.00813377948573137	2.09	P00747 P01024 P01042 P00734
hsa00340 Histidine metabolism	3	13	149	2834	4.39	0.0276072194693599	1.56	P43353 P21397 P30837
hsa00910 Nitrogen metabolism	2	6	149	2834	6.34	0.0358187358419433	1.45	P00915 P00918
hsa04371 Apelin signaling pathway	7	61	149	2834	2.18	0.0387258116740504	1.41	P10301 Q15796 P62879 P61952 Q14344 P50148 O60240
hsa05310 Asthma	3	15	149	2834	3.8	0.0406756033570966	1.39	P13727 P11678 P30273
hsa05150 Staphylococcus aureus infection	6	50	149	2834	2.28	0.0448414576181837	1.35	P00747 P01024 P0C0L5 P12314 P02679 Q92764
hsa04978 Mineral absorption	3	16	149	2834	3.57	0.0481852550196982	1.32	P02787 Q53TN4 P30519

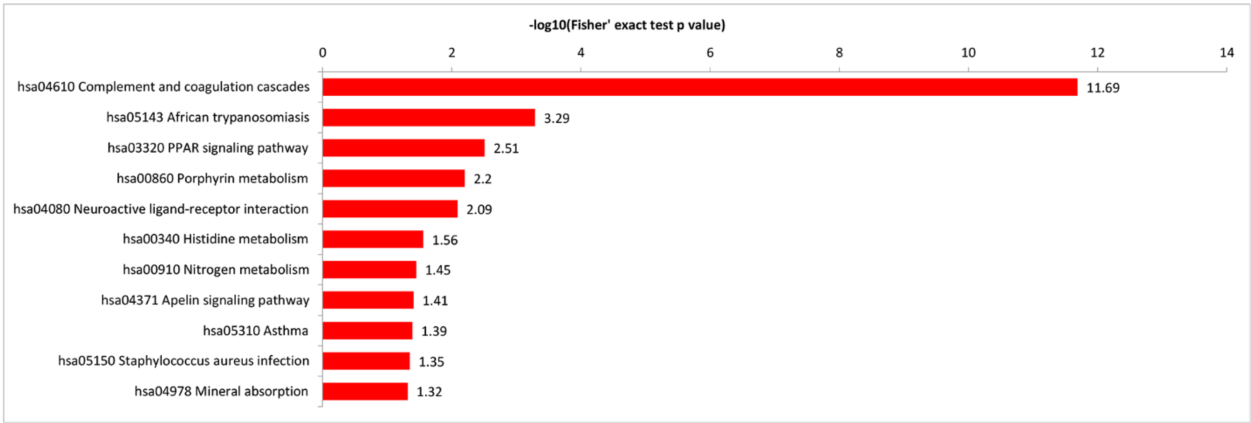

Supplementary Table 12. KEGG pathway enrichment analysis of Q2 protein in psoriasis pathology tissue sections.

KEGG pathway	Mapping	Background	All mapping	All background	Fold enrichment	Fisher's exact test p-value	-log10 (p-value)	Related proteins
hsa04510 Focal adhesion	9	108	73	2834	3.24	0.00152731665240096	2.82	O75116 P49746 Q03135 Q05397 O75369 Q14315 P46108 Q13418 P27361
hsa04012 ErbB signaling pathway	5	41	73	2834	4.73	0.00359370028970315	2.44	P42229 Q05397 P46108 P27361 Q13557
hsa05150 Staphylococcus aureus infection	5	50	73	2834	3.88	0.0084905104569172	2.07	P09871 P02747 P28067 P05156 P19012
hsa05322 Systemic lupus erythematosus	5	52	73	2834	3.73	0.0100084069452076	2	P09871 P02747 P28067 Q16778 P19474
hsa04658 Th1 and Th2 cell differentiation	4	36	73	2834	4.31	0.0128006221416951	1.89	P42229 P28067 P27361 P42226
hsa05220 Chronic myeloid leukemia	4	38	73	2834	4.09	0.0154332453003611	1.81	P42229 Q92466 P46108 P27361
hsa03320 PPAR signaling pathway	4	38	73	2834	4.09	0.0154332453003611	1.81	P33121 P02656 Q13418 Q96Q06
hsa04360 Axon guidance	5	58	73	2834	3.35	0.0156627070676531	1.81	O75116 Q05397 Q13418 P27361 Q13557
hsa05205 Proteoglycans in cancer	7	105	73	2834	2.59	0.0170927890779721	1.77	O75116 Q03135 Q05397 O75369 Q14315 P27361 Q13557
hsa04610 Complement and coagulation cascades	5	64	73	2834	3.03	0.0231418008993157	1.64	P09871 P02747 P01008 P05155 P05156
hsa05133 Pertussis	4	43	73	2834	3.61	0.023434380351256	1.63	P09871 P02747 P05155 P27361
hsa05213 Endometrial cancer	3	25	73	2834	4.66	0.0251186449158574	1.6	Q92466 Q13418 P27361
hsa04659 Th17 cell differentiation	4	44	73	2834	3.53	0.0252873210209065	1.6	P42229 P28067 P27361 P42226
hsa05161 Hepatitis B	5	66	73	2834	2.94	0.0260736942665817	1.58	P42229 Q92466 Q7Z434 P27361 P42226
hsa04935 Growth hormone synthesis, secretion and action	4	48	73	2834	3.24	0.0335697440257818	1.47	P42229 Q05397 P46108 P27361
hsa00790 Folate biosynthesis	2	12	73	2834	6.47	0.0365577557963611	1.44	P35270 Q9NQX3
hsa04261 Adrenergic signaling in cardiomyocytes	4	50	73	2834	3.11	0.0382408441834425	1.42	P09493 Q93084 P27361 Q13557
hsa04062 Chemokine signaling pathway	5	74	73	2834	2.62	0.0401396137908071	1.4	O75116 Q05397 P46108 P27361 Q8TCU6
hsa04217 Necroptosis	5	75	73	2834	2.59	0.0421690998597402	1.38	P42229 Q96EP0 P06737 Q13557 P42226

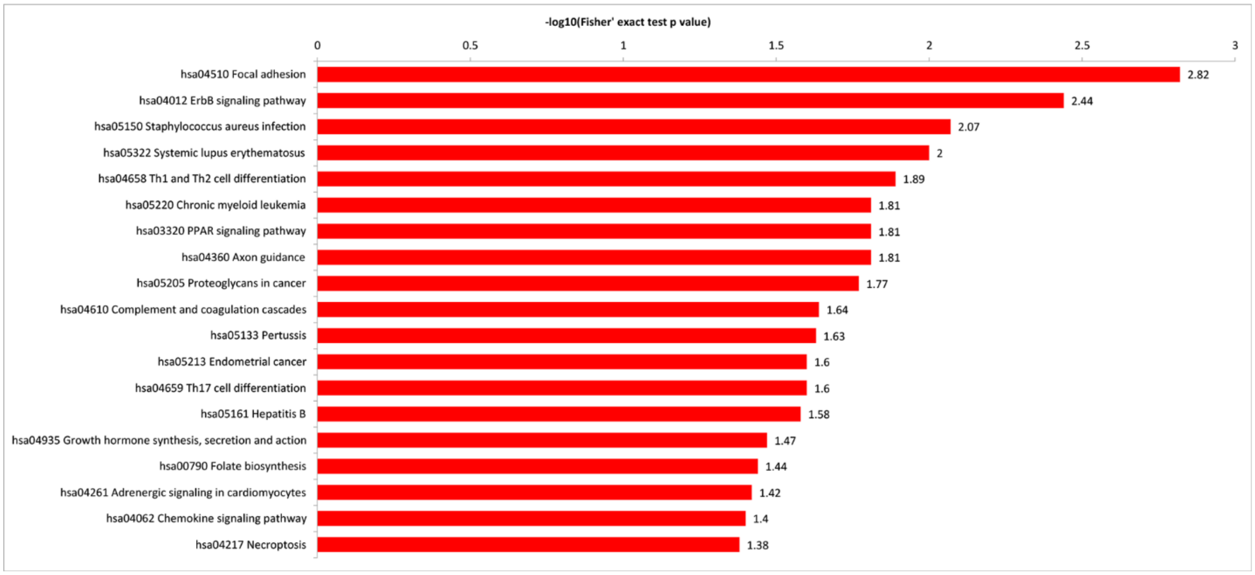
-log10(Fisher' exact test p value)
0
0.5
1
1.5
2
2.5
3
hsa04510 Focal adhesion 2.82
hsa04012 ErbB signaling pathway 2.44
hsa05150 Staphylococcus aureus infection 2.07
hsa05322 Systemic lupus erythematosus 2
hsa04658 Th1 and Th2 cell differentiation 1.89
hsa05220 Chronic myeloid leukemia 1.81
hsa03320 PPAR signaling pathway 1.81
hsa04360 Axon guidance 1.81
hsa05205 Proteoglycans in cancer 1.77
hsa04610 Complement and coagulation cascades 1.64
hsa05133 Pertussis 1.63
hsa05213 Endometrial cancer 1.6
hsa04659 Th17 cell differentiation 1.6
hsa05161 Hepatitis B 1.58
hsa04935 Growth hormone synthesis, secretion and action 1.47
hsa00790 Folate biosynthesis 1.44
hsa04261 Adrenergic signaling in cardiomyocytes 1.42
hsa04062 Chemokine signaling pathway 1.4
hsa04217 Necroptosis 1.38

Supplementary Table 13. KEGG pathway enrichment analysis of Q3 protein in psoriasis pathology tissue sections.

KEGG pathway	Mapping	Background	All mapping	All background	Fold enrichment	Fisher's exact test p-value	-log10 (p-value)	Related proteins
hsa03010 Ribosome	31	106	172	2834	4.82	1.59332166405189E-14	13.8	P62829 P30050 P62906 P46776 P08708 P62701 P82932 P62424 P22090 Q02878 P62888 P05387 P62249 P62857 P83731 P18124 P05388 P62277 P62244 P61353 P63173 P62750 P62847 P84098 P46782 P62851 Q07020 P60866 P62913 P61313 P18077
hsa05171 Coronavirus disease - COVID-19	33	155	172	2834	3.51	3.80877076730991E-11	10.42	P62829 P30050 P62906 Q99836 P46776 P08708 P62701 P62424 P22090 Q02878 P62888 P05387 P62249 P62857 P83731 P18124 P05388 P62277 P62244 O95786 P61353 P63173 P62750 P62847 P84098 P46782 P62851 P40763 Q07020 P60866 P62913 P61313 P18077
hsa03015 mRNA surveillance pathway	9	58	172	2834	2.56	0.00729283044126032	2.14	Q13310 Q9UKF6 P35637 O43809 P62495 Q86U42 P62136 P51003 Q86V81
hsa00760 Nicotinate and nicotinamide metabolism	4	16	172	2834	4.12	0.0134076491169586	1.87	P00491 P49902 Q06278 P43490
hsa00970 Aminoacyl-tRNA biosynthesis	5	27	172	2834	3.05	0.0210625411587651	1.68	Q9Y285 Q15046 P12081 P26639 P49591
hsa03040 Spliceosome	13	117	172	2834	1.83	0.0279485728569399	1.55	Q9BWJ5 O14776 Q9Y2W2 Q86XP3 P35637 P55769 O60828 Q86V81 P62304 P09651 O15042 Q96FV9 Q9Y3C6
hsa00750 Vitamin B6 metabolism	2	5	172	2834	6.59	0.0324291844550689	1.49	Q9Y617 Q06278
hsa03050 Proteasome	6	41	172	2834	2.41	0.0349861796654851	1.46	Q9UL46 P51665 Q06323 P62195 O14818 O00487
hsa03008 Ribosome biogenesis in eukaryotes	7	52	172	2834	2.22	0.0354229688789109	1.45	Q8NI36 Q9Y3A5 Q8IWA0 Q8N5L8 Q9H0D6 P55769 P62826
hsa00240 Pyrimidine metabolism	5	33	172	2834	2.5	0.0462387361397857	1.33	P00491 P19971 P49902 Q9H773 P15531
hsa04623 Cytosolic DNA-sensing pathway	4	23	172	2834	2.87	0.0468842339601234	1.33	P62875 O95786 Q9ULZ3 Q14116

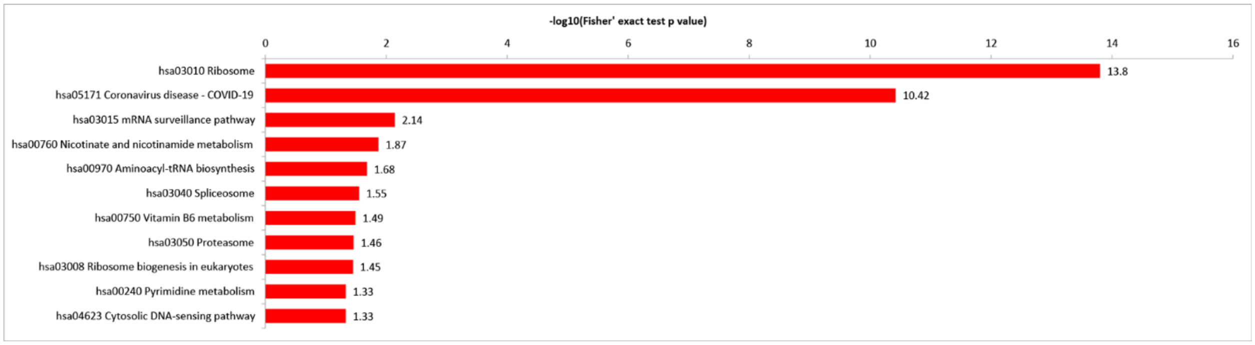
-log10(Fisher' exact test p value)
0
2
4
6
8
10
12
14
16
hsa03010 Ribosome
hsa05171 Coronavirus disease - COVID-19
hsa03015 mRNA surveillance pathway
hsa00760 Nicotinate and nicotinamide metabolism
hsa00970 Aminoacyl-tRNA biosynthesis
hsa03040 Spliceosome
hsa00750 Vitamin B6 metabolism
hsa03050 Proteasome
hsa03008 Ribosome biogenesis in eukaryotes
hsa00240 Pyrimidine metabolism
hsa04623 Cytosolic DNA-sensing pathway
13.8
10.42
2.14
1.87
1.68
1.55
1.49
1.46
1.45
1.33
1.33

Supplementary Table 14. KEGG pathway enrichment analysis of Q4 protein in psoriasis pathology tissue sections.

KEGG pathway	Mapping	Background	All mapping	All background	Fold enrichment	Fisher's exact test p-value	-log10 (p-value)	Related proteins
hsa03008 Ribosome biogenesis in eukaryotes	11	52	101	2834	5.94	1.21977150331164E-06	5.91	Q9UBU9 Q969X6 Q15061 Q8TED0 O60832 Q9BVP2 Q9BZE4 Q96G21 Q9ULX3 Q9H583 Q99575
hsa03010 Ribosome	11	106	101	2834	2.91	0.00112242705906763	2.95	Q9Y3D3 P40429 P52815 P39019 P18621 P63220 Q6P5R6 P62273 P39023 P15880 P62081
hsa05171 Coronavirus disease - COVID-19	13	155	101	2834	2.35	0.00289190376123779	2.54	Q9Y6K5 P40429 P39019 P18621 P63220 O14920 P20591 Q6P5R6 P62273 P39023 P15880 P19174 P62081
hsa04657 IL-17 signaling pathway	5	33	101	2834	4.25	0.00558227404580776	2.25	Q86SG5 O14920 P31151 P05109 P06702
hsa00790 Folate biosynthesis	3	12	101	2834	7.01	0.00764639209240929	2.12	P09417 O60218 Q92820
hsa05418 Fluid shear stress and atherosclerosis	7	67	101	2834	2.93	0.00890393646028822	2.05	O14920 O15217 Q9NZT1 P16284 P27482 P35052 Q14145
hsa01523 Antifolate resistance	3	13	101	2834	6.48	0.00968582937230774	2.01	O14920 Q92820 P33527
hsa04744 Phototransduction	2	5	101	2834	11.22	0.0117229388047486	1.93	Q9NZT1 P27482
hsa04070 Phosphatidylinositol signaling system	5	40	101	2834	3.51	0.012711195462184	1.9	Q9NZT1 P27482 O14732 Q13613 P19174
hsa04060 Cytokine-cytokine receptor interaction	3	15	101	2834	5.61	0.0146322830649248	1.83	Q9UBH0 P18510 Q9NZH8

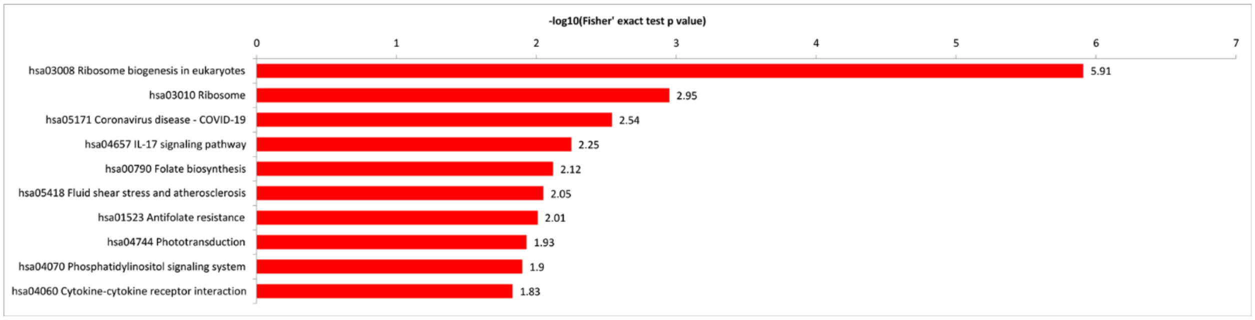

Supplement: Supplementary Tables 10-14 [file aging-16-205478-s011.pdf]
